# Advancing homogeneous catalysis for parahydrogen-derived hyperpolarisation and its NMR applications†

**DOI:** 10.1039/d2sc00737a

**Published:** 2022-03-22

**Authors:** Ben. J. Tickner, Vladimir V. Zhivonitko

**Affiliations:** NMR Research Unit, Faculty of Science, University of Oulu P.O. Box 3000 Oulu 90014 Finland ben.tickner@alumni.york.ac.uk vladimir.zhivonitko@oulu.fi; Department of Chemical and Biological Physics, Faculty of Chemistry, Weizmann Institute of Science Rehovot 7610001 Israel

## Abstract

Parahydrogen-induced polarisation (PHIP) is a nuclear spin hyperpolarisation technique employed to enhance NMR signals for a wide range of molecules. This is achieved by exploiting the chemical reactions of parahydrogen (para-H_2_), the spin-0 isomer of H_2_. These reactions break the molecular symmetry of para-H_2_ in a way that can produce dramatically enhanced NMR signals for reaction products, and are usually catalysed by a transition metal complex. In this review, we discuss recent advances in novel homogeneous catalysts that can produce hyperpolarised products upon reaction with para-H_2_. We also discuss hyperpolarisation attained in reversible reactions (termed signal amplification by reversible exchange, SABRE) and focus on catalyst developments in recent years that have allowed hyperpolarisation of a wider range of target molecules. In particular, recent examples of novel ruthenium catalysts for *trans* and *geminal* hydrogenation, metal-free catalysts, iridium sulfoxide-containing SABRE systems, and cobalt complexes for PHIP and SABRE are reviewed. Advances in this catalysis have expanded the types of molecules amenable to hyperpolarisation using PHIP and SABRE, and their applications in NMR reaction monitoring, mechanistic elucidation, biomedical imaging, and many other areas, are increasing.

## Abstract 2

We detail recent advances in homogeneous catalysts for deriving enhanced NMR signals using parahydrogen. Growing applications of this catalysis in mechanistic elucidations, mixture analysis, and biomedical imaging are also discussed.

## Graphical abstract

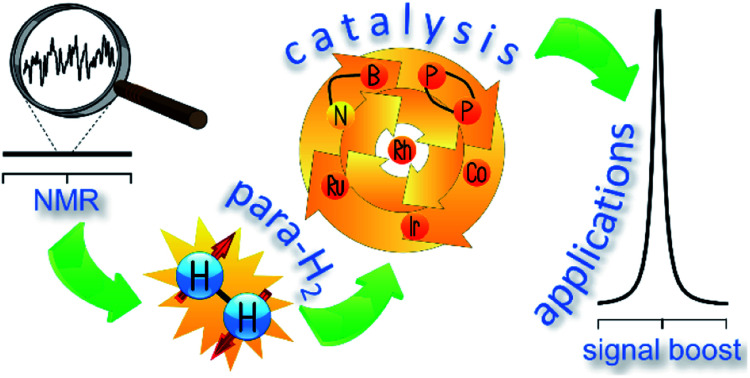

## 1. Introduction

Nuclear Magnetic Resonance (NMR) spectroscopy and imaging are widely known methods that examine nuclear spins within molecules and materials. Consequently, they provide important structural, dynamic, and spatial information about matter without using ionising radiation that can often destroy the samples under examination. Moreover, they can be applied to study any state of matter: gas, liquid, or solid. For these reasons, NMR-based techniques have become a method-of-choice for many scientists to interrogate the structure and dynamic properties of small molecules, proteins, catalytic and functional materials, tissues and many other systems.^1^ Despite these advantages, they are considered relatively insensitive as concentrated samples (≥mM concentrations) are often required to get strong enough signals to draw any conclusions. This insensitivity stems from the small population differences across closely spaced nuclear spin energy levels which is dictated by the Boltzmann distribution under standard thermal equilibrium conditions. This is evident from the tiny fraction of nuclear spins (*e.g.*, 1 in every 32 000 ^1^H nuclei at 9.4 T) that effectively contribute to a detected NMR signal. The sensitivity issue becomes even more pronounced for nuclei that possess a smaller gyromagnetic ratio (such as ^13^C, ^15^N, ^19^F, ^31^P and many others) and at lower magnetic fields, as nuclear spin states become closer in energy. This is usually mitigated by using concentrated samples and/or time-consuming signal averaging to generate spectra of sufficient signal-to-noise ratio (SNR).

Great effort is focussed towards instrumental and methodological developments that improve the sensitivity of NMR.^2,3^ As a result, approaches collectively known as hyperpolarisation have arisen from these efforts. Hyperpolarisation refers to creation of a non-equilibrium nuclear spin state with dramatically larger population differences between the energy levels relative to those for thermally polarised samples (*i.e.*, those coming from a Boltzmann distribution). NMR signals can be orders of magnitude more intense as a consequence.^3^ Hyperpolarisation is an active topic of research as it can significantly reduce experiment times since large numbers of repetitions are no longer required to generate discernible NMR signals. The enhanced NMR signals provided by hyperpolarisation have advantages for many applications such as detecting reaction intermediates and reaction monitoring, mechanistic elucidations, analysis of low concentration mixtures, and metabolic imaging.^4–7^

Several hyperpolarisation techniques are known to derive strong non-equilibrium nuclear spin polarisation *via* various sources.^4^ For instance, Spin Exchange Optical Pumping (SEOP)^8^ and dissolution Dynamic Nuclear Polarisation (DNP)^9^ utilize circularly polarised light and polarisation of electron spins, respectively, to hyperpolarise nuclear spins. To a great extent, these methods are underpinned by advanced physics and have both been known for at least the last 65 years.^4^ In this review, we focus on hyperpolarisation techniques that are much more chemical by nature since they utilise catalytic transformations of parahydrogen (para-H_2_), the spin-0 isomer of H_2_. Para-H_2_ serves as both a chemical reagent and the source of hyperpolarisation at the same time. Methods that exploit para-H_2_ spin order as a source of hyperpolarisation are collectively termed as ParaHydrogen-Induced Polarisation (PHIP) and have been developed since the 1980s.^6,10–12^ They provide a low-cost route to hyperpolarise target molecules because expensive equipment is not required and the para-H_2_ feedstock is cheap and easy to produce.^13^

The para-H_2_ molecule is NMR silent (*i.e.* it does not give any NMR signal). However, breaking the symmetry of para-H_2_ in a chemical reaction can lead to a dramatic enhancement of NMR signals of the reaction products.^14,15^ This chemistry underpins the PHIP effect and can be hugely beneficial for the detection of low concentration (≪1 mM) or short-lived molecules (<1 minute) that might be challenging to observe using conventional NMR with thermal polarisation. Herein, we discuss various types of chemical reactions that can break para-H_2_ symmetry and give rise to PHIP. These reactions are often catalysed by transition metal complexes, and we discuss the role these species play in achieving PHIP. In general, many researchers are working to increase the substrate scope, efficiency, conversion rates, lifetime of hyperpolarised states, solvent tolerability, and many other properties of these catalysts which are directly linked to the NMR signal enhancements that can be produced. Therefore, we focus on recent advances in homogeneous catalyst design which have allowed the hyperpolarisation of a wider range of molecules with greater efficiency using PHIP and related SABRE^16,17^ effects. We also give some key examples of the tremendous value these novel catalysts have in the areas of mixture analysis, mechanistic elucidation, reaction monitoring, and biomedical imaging. In addition to metal complexes, recent emerging metal-free catalysts are also discussed.

## 2. Para-H_2_: properties, production and PHIP

In this review we describe the properties and production of para-H_2_ only briefly and refer the interested reader to other works^6,17–19^ for specific details. Generally, at room temperature dihydrogen gas (H_2_) exists as a mixture of *ortho* and *para* nuclear spin isomers quite accurately in a 3 : 1 ratio, and conversion between these isomers is slow (on the order of days and even longer) due to the symmetry disallowed nature of this transition.^18^ H_2_ can be enriched in the lower energy para isomer (para-H_2_) *via* interactions with a metal centre or a paramagnetic material (catalyst) at low temperatures.^20–22^ Para-H_2_ enrichments of up to 50% can occur at liquid nitrogen temperatures (77 K) and even higher values of 98% can be achieved at 28 K.^5^ This enrichment survives removal of the spin interconversion catalyst and return to ambient temperature,^13,20,22^ and it is important since analogous PHIP reactions with hydrogen gas thermally equilibrated at or near room temperature does not typically produce NMR signal enhancements.

**Fig. 1:**
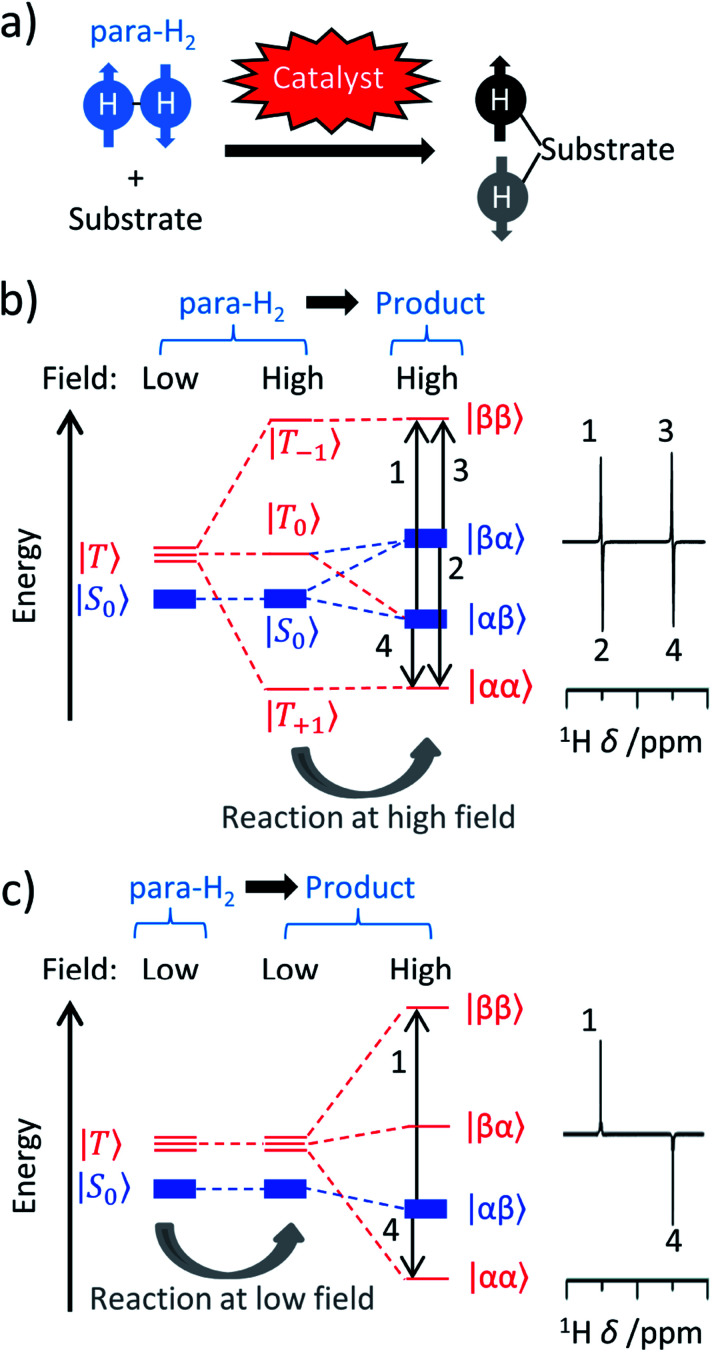
(a) Parahydrogen is ‘NMR silent’ and can only be observed by NMR if its symmetry can be broken in a pairwise reaction, often catalysed by a metal centre. Products containing ^1^H nuclei that were originally located in para-H_2_ can exhibit NMR signals orders-of-magnitude more intense than would be recorded under ‘thermal’ (*i.e.* Boltzmann) controlled conditions. (b) Under PASADENA conditions, the para-H_2_ symmetry-breaking process in a chemical reaction occurs at the spectrometer (high) field and results in population of both the |αβ〉 and |βα〉 nuclear spin energy levels of the product. (c) Under ALTADENA conditions the reaction occurs at Earth's (low) field outside the spectrometer before insertion into the spectrometer for NMR detection. As a result, only one of the |αβ〉 or |βα〉 levels of the product are populated. In this depiction the |αβ〉 state is shown lower in energy and populated. Note that the two protons in the product are assumed to form a weakly coupled AX spin system and the energies are not shown to scale. NMR spectra may show a different appearance when recorded using different flip angles. In these depictions, the population of each state is indicated by the thickness of the line.

Para-H_2_ has only one nuclear spin manifold and its wavefunction is denoted as (|αβ〉 − |βα〉)/
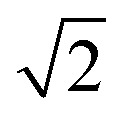
 or |S_0_〉 (nuclear spin singlet). Ortho-H_2_ has a nuclear spin degeneracy of three in the absence of a magnetic field, and these states can be described as |αα〉, (|αβ〉 + |βα〉)/
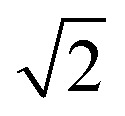
, and |ββ〉, or by using the notation |T_+1_〉, |T_0_〉, and |T_−1_〉 (nuclear spin triplet), respectively. In this notation, α and β are spin-up and spin-down states of individual nuclei in H_2_ molecules. As para-H_2_ has a nuclear spin quantum number of 0, it does not respond with a signal to the radiofrequency excitation applied during NMR pulse sequences and is often referred to as being ‘NMR silent’. The symmetry of para-H_2_ must be broken to allow the pair of ^1^H nuclei to become observable using ^1^H NMR. This can be achieved in a catalytic process in which, *e.g.*, the ^1^H nuclei of the para-H_2_ molecule are incorporated into a target molecule (Fig. 1a).^9,24,25^ These newly introduced protons can exhibit dramatically enhanced ^1^H NMR signals. The nuclear hyperpolarisation, however, is typically short-lived and decays reaching the thermal equilibrium level due to nuclear spin relaxation which is typically characterised by the time taken to establish thermal longitudinal magnetisation, *T*_1_.

A typical requirement for such reactions to yield strong PHIP effects is that catalysed incorporation of para-H_2_ into the target substrate must occur in a pairwise manner. In other words, both introduced ^1^H nuclei should originate from the same para-H_2_ molecule for them to transfer the two-spin correlation (nuclear spin order) inherited from para-H_2_ to the substrate. In cases of non-pairwise addition, the spin correlation is not transferred to the substrate and, as a consequence, PHIP effects will not be observed. At the same time, PHIP can be observed for catalytic intermediates if initial para-H_2_ activation by the catalyst active centre is pairwise, although the full cycle can be non-pairwise. Based on this possibility, effects such as oneH-PHIP^23–28^ can be observed wherein only one of the para-H_2_ originating protons ends up in the hyperpolarised product. There are also reactions in which molecules can become hyperpolarised without chemical incorporation of para-H_2_ into the target structures, as takes place in SABRE.^16,17,29^ These processes still contain a pairwise symmetry-breaking step in the catalytic cycle and rely on other means (*e.g.*, level anticrossings, rf pulsing) to transfer the polarisation or the spin order to the target sites. Therefore, an understanding of the catalysis underpinning these reactions is clearly vital to rationalise and account for these differing effects.

The appearance of PHIP NMR spectra can differ if the catalytic reaction takes place directly at the high magnetic field of the spectrometer (while the sample is inside the magnet) or outside the magnet at the Earth's magnetic field (sometimes referred to as ‘low’ field) before the sample is transferred to the spectrometer for ‘high’ field NMR detection.^14,18,30,31^ For instance, Fig. 1b and c shows schematically the influence of these two different (spectrometer and Earth's) fields on the populations of the nuclear spin states of para-H_2_ and the resultant reaction product(s) in the case when the para-H_2_ originating protons form a weakly coupled AX spin system at high field. These regimes have been termed Parahydrogen And Synthesis Allow Dramatically Enhanced Nuclear Alignment (PASADENA)^14^ for symmetry-breaking at high field and Adiabatic Longitudinal Transport After Dissociation Engenders Nuclear Alignment (ALTADENA)^32^ for symmetry-breaking at low field. Hyperpolarisation created under ALTADENA and PASADENA conditions may display different ^1^H NMR signal shapes (Fig. 1b and c), although both approaches can be described more generally as PHIP.

## 3. Hyperpolarised metal dihydrides: ligation and oxidative addition of para-H_2_

There are different types of chemical reactions that can break the symmetry of para-H_2_ in a pairwise fashion. Perhaps the simplest of these is ligation of para-H_2_ to a metal centre to form an η^2^-H_2_ complex. The implications of this for catalysis are that the improvement in NMR sensitivity provided by PHIP can make detection of η^2^-H_2_ complexes feasible. An excellent example is the indirect detection of a [Ni(η^2^-H_2_)(L-κ^2^*P,P'*)_2_]^2+^
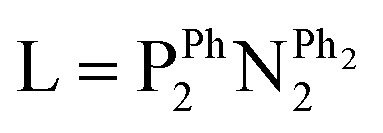
 complex that is impossible to discern using standard thermally-polarised ^1^H NMR measurements.^33^ The short-lived nature of η^2^-H_2_ species usually requires advanced NMR methods (such as Chemical Exchange Saturation Transfer (CEST) and partially negative line effect) to detect them indirectly.^33–35^

**Fig. 2:**
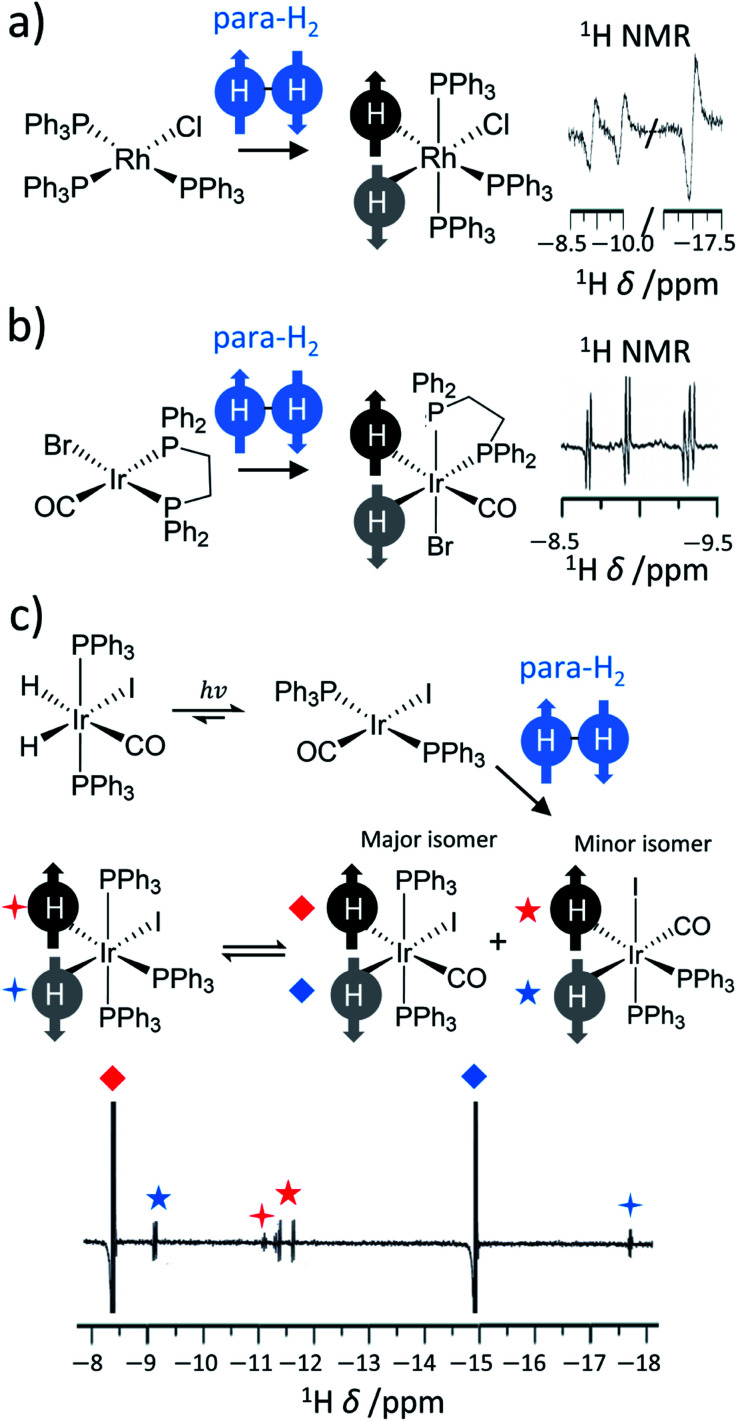
Examples of symmetry-breaking oxidative addition reactions. Formation of hyperpolarised (a) [RhCl(H)_2_(PPh_3_)_3_] upon para-H_2_ addition to [RhCl(PPh_3_)_3_], (b) [IrBr(H)_2_CO(PPh_2_(CH_2_)_2_PPh_2_)] following para-H_2_ addition to [IrBrCO(PPh_2_(CH_2_)_2_PPh_2_)] and (c) [IrCO(H)_2_I(PPh_3_)_2_] upon para-H_2_ addition to [IrCOI(PPh_3_)_2_]. Enhanced hydride NMR signals for a minor geometric isomer of [IrCO(H)_2_I(PPh_3_)_2_] are also visible as para-H_2_ addition can occur to both symmetry axes of [IrCOI(PPh_3_)_2_]. Hyperpolarised hydride NMR signals for [Ir(H)_2_I(PPh_3_)_3_], which is formed from CO loss from [IrCO(H)_2_I(PPh_3_)_2_], are also discerned. Adapted from (a) ref. 14 (b) ref. 31 and (c) ref. 36.

Further reaction of η^2^-H_2_ complexes to form metal dihydrides can result in PHIP effects as oxidative addition of para-H_2_ to a metal centre also breaks para-H_2_ symmetry.^11,36–39^ Activation of dihydrogen by transition metal centres has been known since the 1960s.^40,41^ This typically occurs *via* a concerted mechanism where the newly introduced groups are located in a *cis* relationship.^9,10^ These examples provide a fertile playground for PHIP if this activation is pairwise and faster than nuclear spin relaxation. Early examples in the late 1980s were dihydride complexes of Rh, Ir and Ru.^14,15,30,42^ Many of these are short-lived intermediates in catalytic processes^27,43–45^ (such as hydrogenation or hydroformylation), whereas others are stable hydride species whose ligand exchange processes may be reversible or even light-activated.^36,46,47^ For example, one of the first reported examples of PHIP was the activation of para-H_2_ by [RhCl(PPh_3_)_3_] to form [RhCl(H)_2_(PPh_3_)_3_] in which the ^1^H NMR signals of its hydride ligands were significantly enhanced (Fig. 2a).^14^ Related examples around this time showed hyperpolarised ^1^H NMR signals for the hydride ligands of [IrBr(H)_2_CO(PPh_2_(CH_2_)_2_PPh_2_)], which forms upon oxidative addition of para-H_2_ to [IrBrCO(PPh_2_(CH_2_)_2_PPh_2_)] and is the first step in the hydrogenation of alkynes to alkenes using this catalyst (Fig. 2b).^31^ In these contexts, the enhanced ^1^H NMR signals for the hydride sites of transition metal complexes are extremely useful for enabling their detection, particularly in cases where the metal complex may be at low concentration. Namely, hydride NMR signals for catalytic intermediates such as [Rh(H)_2_Cl(PPh_3_)_2_(styrene)], which is formed following phosphine loss from [RhCl(H)_2_(PPh_3_)_3_] and subsequent alkene coordination, can be made visible using PHIP.^45^ This intermediate is involved in the hydrogenation of alkenes and the detection of such species can be important in determining reaction mechanisms.^7,10,11^

In many of these mechanisms, activation of para-H_2_ is irreversible and, as a consequence, PHIP effects can only be observed over a narrow time window in which reactivity is occurring and nuclear spin relaxation is yet to dominate. Many catalysts, particularly those based on iridium complexes, have been shown to reversibly activate para-H_2_ and therefore enhanced hydride signals can be observed at will upon reaction with fresh para-H_2_.^29,35,48^ This is particularly useful in mechanistic studies involving iridium dihydride complexes^38^ and the detection of low concentrations of metal complexes for which enhanced hydride resonances may be diagnostic of particular ligands in complex mixtures.^38,49–51^ Reactivity of this type is exploited in SABRE as enhanced hydride polarisation can be transferred to other sites in the catalyst (this is discussed in more detail in Section 7).^29^

Other catalysts utilise photochemistry to promote ligand loss pathways and produce enhanced hydride NMR signals.^52^ For example, a [IrCOI(PPh_3_)_2_] complex can be formed from the light-induced reductive elimination of hydrogen from [IrCO(H)_2_I(PPh_3_)_2_]. The original [IrCO(H)_2_I(PPh_3_)_2_] can be reformed from [IrCOI(PPh_3_)_2_], albeit in a hyperpolarised state, by oxidative addition of para-H_2_ (Fig. 2c). In this example, PHIP enables the formation of metal hydrides over millisecond timescales to be monitored and kinetic rate constants for this process to be determined: feats that are impossible to achieve using thermal NMR polarisation due to the need to perform time consuming signal averaging.^52^

As the systems that undergo homolytic dihydrogen splitting have increased rapidly in recent years, an analogous increase in PHIP catalysts has accompanied this. Nowadays, metal catalysts based on Pd,^47,53–56^ Co^26,27,57–59^ and Pt^23^ have all been reported to break para-H_2_ symmetry *via* oxidative addition. We also note that metal clusters^11^ and heterogeneous supported catalysts^60^ have emerged as valuable catalysts that can activate para-H_2_ giving rise to PHIP and SABRE effects. The heterogeneous systems have been described in detail elsewhere.^5,61,62^

## 4. Metal-free PHIP: heterolytic para-H_2_ splitting and beyond

**Fig. 3:**
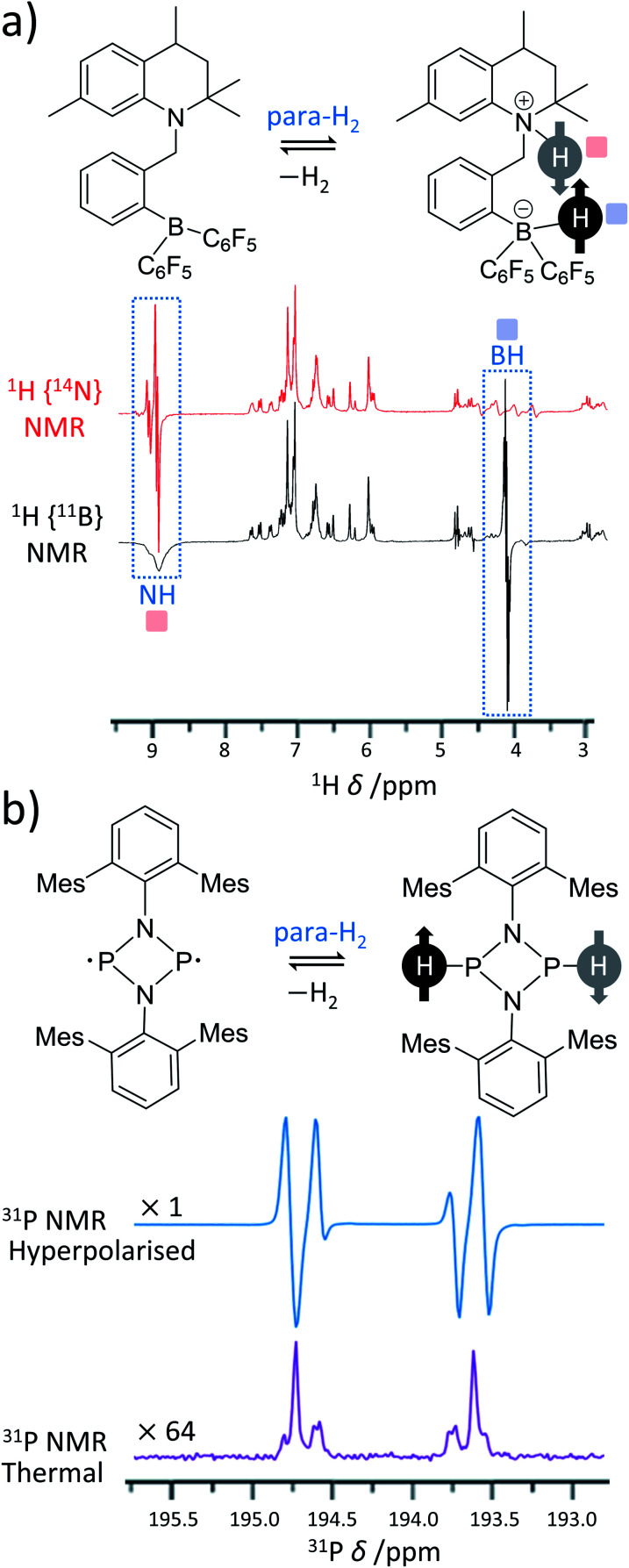
Examples of PHIP in metal-free para-H_2_ activations. (a) *ansa*-Aminoboranes can activate para-H_2_ using a N–B frustrated Lewis pair centre. For example, ^14^N- and ^11^B-decoupled ^1^H NMR spectra acquired using QCAT *ansa*-aminoborane are presented. (b) Pnictogen biradicaloids can also activate para-H_2_ and produce PHIP. For instance, a P–P biradicaloid pair where Mes = 2,4,6-trimethylphenyl gives the presented ^31^P NMR spectrum. For comparison, the thermal ^31^P spectrum expanded vertically by a factor of 64 is also shown in the figure. Adapted from (a) ref. 66 and (b) ref. 67.

Over recent years, many metal-free catalysts have been developed to activate para-H_2_. Most of these systems are based on the use of frustrated Lewis pairs (FLPs)^63^ and can split para-H_2_ heterolytically in a way that also produces enhanced NMR signals for sites within the FLP.^64–69^ The heterolytic activation occurs in a pairwise manner for hyperpolarised reaction products to be observed and can provide valuable insight into the mechanism of H_2_ activation by FLPs. These effects were first demonstrated using *ansa*-aminoborane FLPs such as 1-{2-[bis(pentafluorophenyl)boryl]benzyl}-2,2,4,7-tetramethyl-1,2,3,4-tetrahydroquinoline (QCAT) (Fig. 3a).^66^ Activation of para-H_2_ by QCAT leads to formation of a QCAT-H_2_ adduct in which the ^1^H NMR signals for those nuclei originating from para-H_2_ are enhanced by up to 30-fold. Polarisation transfer from these sites to ^11^B nuclei was achieved using an INEPT-type pulse sequence and yielded a 10-fold enhancement in ^11^B NMR signal intensity. Since then, a wider range of *ansa*-aminoboranes have been used for metal-free PHIP, including those activating para-H_2_ under ambient temperatures in a reversible fashion.^65^ Spontaneous polarisation transfer from para-H_2_ derived ^1^H nuclei to ^11^B and ^15^N sites in a 7 T spectrometer is also possible with significant 350-fold signal enhancements recorded for ^15^N sites of the FLP.^64^ Activation of para-H_2_ using FLPs based on P–C^68^ and P–Sn^70^ have also been documented.

In addition to FLPs, P–P^67,69^ and As–P^69^ biradicaloids, an alternative class of metal-free para-H_2_ activators, have also been used in PHIP with up to 2000-fold signal enhancements for ^31^P sites at 9.4 T. In this case, the activation is homolytic in nature leading to the formation of hyperpolarised biradicaloid-H_2_ adducts. In addition to enhanced para-H_2_-derived ^1^H sites, the hyperpolarisation of ^31^P sites could be observed either spontaneously or by using rf pulsing to transfer polarisation from ^1^H to ^31^P, depending on the structure of the biradicaloid (Fig. 3b). Generally, P–P, As–P and As–As biradicaloids containing four- and five-membered rings were shown to activate para-H_2_, in many cases reversibly.^69^ This reversibility allowed the observation of hyperpolarised starting para-H_2_ metal-free activators (FLPs^64^ or biradicaloids^69^) without actual modification of their structures and demonstrates features of a metal-free SABRE effect. In these cases, PHIP has been used as a great mechanistic tool to study para-H_2_ activation by such systems.

While current examples of metal-free systems for para-H_2_ activation have been reported in organic solvents such as toluene-*d*_8_,^66,69^ dichloromethane-*d*_2_,^64,65^ and bromobenzene-*d*_5_,^68^ they may in the future provide a promising route for PHIP in aqueous solvents without the use of toxic metal catalysts. Generally, metal-free PHIP using FLPs offers an alternative route to break para-H_2_ symmetry that does not rely on a transition metal complex for traditional oxidative addition. Homolytic H_2_ activation typically involves complex orbital overlap effects.^11^ Repulsive interactions between filled orbitals on the metal centre and the H_2_ ligand can be a barrier to oxidative addition that must be overcome. For some metal complexes, this barrier can be so large that heterolytic splitting of H_2_ becomes feasible.^71^ Therefore, examples of metal-catalysed heterolytic para-H_2_ addition^34,71,72^ to generate enhanced NMR signals for reaction products may emerge in the future.

## 5. Hydrogenation reactions

### 5.1 Transition metal catalysed alkyne and alkene hydrogenation

**Fig. 4:**
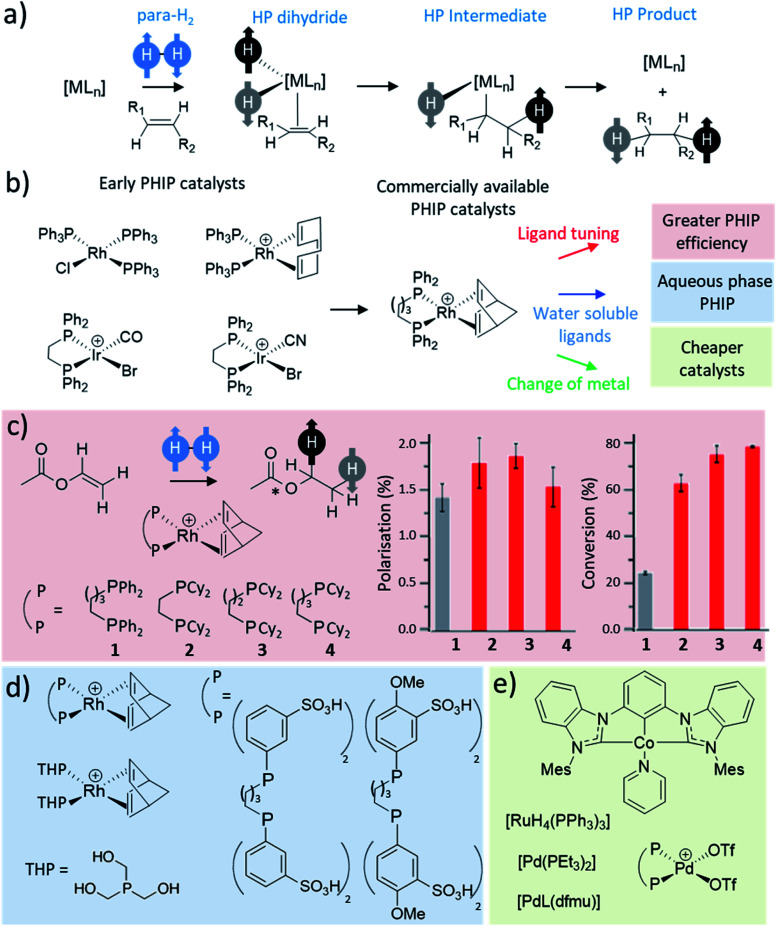
(a) Depiction of the general process of a pairwise hydrogenation reaction. Oxidative addition is the primary step that breaks the symmetry of para-H_2_. Subsequent steps such as substrate coordination, hydride migration, and product elimination, yield a hydrogenated product with enhanced ^1^H NMR signals. (b) Examples of catalysts reported for early PHIP studies^14,30,31,42,45^ with commercial catalysts commonly used for PHIP today. (c) Example hydrogenation of vinyl acetate to form ethyl acetate, which was used to study the effect of Rh catalyst on factors such as ^13^C polarisation level and conversion to the product. Adapted from ref. 90. (d) Example water-soluble catalysts reported to produce PHIP in aqueous solutions.^44,98^ (e) Representative examples of ruthenium,^15^ palladium^47,54^ and cobalt^59^ catalysts used for PHIP.

Many catalytic processes involve H_2_ activation as an initial step, including metal-catalysed hydrogenation or hydroformylation. Early PHIP studies that involved para-H_2_ activation by square planar group 9 precatalysts of rhodium^30,42^ and iridium^31,73,74^ were accompanied by transfer of these newly introduced hydrides to an alkyne or alkene (Fig. 4a). If this reaction occurs rapidly relative to the relaxation rate of the hyperpolarised metal dihydride then enhanced polarisation may survive transfer into the product.^14,30,31,45^ Early PHIP studies involved hydrogenation of simple alkenes and alkynes such as styrene and phenylacetylene,^15,30,75^ although in more recent years PHIP has been extended to significantly more complex molecules including amino acid derivatives^76^ and even complex synthetic oligopeptides which have been of use to probe protein–ligand interactions.^77–82^ Heteronuclear polarisation within the transition metal hydrogenation catalysts, or hydrogenated reaction products, can also be achieved following either dedicated pulse sequences,^74,76,83–85^ spontaneous low field transfer,^86^ or field cycling.^87–89^

The most commonly used catalysts for these PHIP hydrogenation reactions remain based on rhodium, and to a lesser extent iridium (Fig. 4b), although there were early examples of less well-defined systems using ruthenium^15^ to achieve hyperpolarised signals for hydrogenated products. Rapid hydrogenation is required to observe PHIP effects before nuclear spin relaxation destroys the hyperpolarisation. Therefore, optimisation of these catalysts is essential as the level of hyperpolarisation achieved strongly depends on factors such as hydrogenation rate.^90^ Significant efforts have been directed at the optimisation of catalysts for standard hydrogenation reactions, which usually focus on improving the catalyst turnover, reactivity and selectivity. Such efforts are not typically applied to the optimisation of hydrogenation catalysts for PHIP reactions, which often use commercially available Rh catalysts (such as [Rh(diene)(dppb)][BF_4_], diene = norbornadiene or 1,5-cyclooctadiene) that are sometimes incapable of completely hydrogenating a target molecule in just a few seconds without the use of high H_2_ pressure.^91^ In PHIP reactions, the presence of paramagnetic impurities, catalyst degradation products, resting states, or transient intermediates are likely to relax hyperpolarised molecules and destroy the PHIP effect. As soon as para-H_2_ is ligated to a metal centre its symmetry is broken and the spin order can then be lost before further transfer of the para-H_2_ originating protons. Therefore, unwanted polarisation loss will depend on relaxation and the lifetimes of catalyst-bound para-H_2_ species, of which there may be many in the catalytic cycle. These factors will play a role in the polarisation transfer efficiency of PHIP and are additional factors that are not typically considered in traditional hydrogenation catalysis with thermally equilibrated H_2_.

Therefore, the roles of catalyst structure and concentration on hyperpolarisation efficiency were studied for [Rh(COD)(BINAP)][BF_4_] and [Rh(COD)(dppb)][BF_4_] catalysts in butyl acrylate hydrogenation using para-H_2_.^92^ The authors defined a parameter called polarisation transfer efficiency (PTE) which quantified the ratio of the experimental and the idealised hyperpolarisation that could be achieved for the product (butyl propanoate) in an ensemble of product molecules; it depended on measurables such as relaxation and hydrogenation rate. For [Rh(COD)(BINAP)][BF_4_], PTE increased as the catalyst concentration is lowered, and generally increased as reaction time is increased, which was related to a drop in hydrogen pressure as the reaction proceeds. The authors suggested that this effect is likely related to a shortened residence time of the para-H_2_ molecule in the coordinated state on the metal centre when the alkene/hydrogen ratio decreases.^92^ The shorter residence can favour high PTE since the time for relaxation of the para-H_2_ spin order is reduced. The PHIP efficiency was stronger at higher magnetic fields (14 T compared to 7 T)^92^ where the *T*_1_ relaxation time of dihydrides is typically longer.^93^ The change of PTE as a function of hydrogenation reaction time appeared different demonstrating rise and fall behavior when the [Rh(COD)(dppb)][BF_4_] catalyst was used which highlights the importance the catalyst can have on the efficiency of the PHIP process. The reaction time, magnetic field, catalyst identity and concentration, all influence the efficiency of PHIP and must be carefully considered in conjunction with the rate, turnover and yield usually addressed in studies of traditional hydrogenation systems.

Some studies have sought to improve the Rh complexes used in PHIP by variation of ligands in the coordination sphere. A series of [Rh(norbornadiene)(bisphosphine)][BF_4_] catalysts (1–4) were used to hydrogenate the example substrate vinyl acetate.^90^ A variety of different bisphosphines ligands were screened and their effect on hydrogenation kinetics and the magnitude of PHIP NMR signal enhancements were recorded (Fig. 4c). This study demonstrated that highly electron-donating bisphosphines with dicyclohexylphosphine groups (2–4) gave higher catalytic activity with 1.8–2.0 times higher turnover frequencies compared to the standard commercially available [Rh(norbornadiene)(dppb)][BF_4_] (1).^90^ The initial precatalyst activation involves the loss of the norbornadiene ligand and its replacement with two solvent molecules (in this case, methanol). This activation step was faster using 2–4 as the rate of norbornadiene ligand loss was 3.3–4.8 times faster than the standard Rh system.^90^ Similarly, hydrogenation was also faster in the case of the modified catalysts which in turn gave rise to higher NMR signal enhancements in PHIP experiments. The reason for this higher catalytic activity is yet unconfirmed with the mechanism of this transformation not yet completely understood. The active [Rh(solvent)_2_(diphosphine)][BF_4_] catalyst can coordinate substrate before irreversible oxidative addition of hydrogen.^94^ However, routes in which hydrogen activation occurs before substrate coordination have also been proposed.^95^ Further studies of this nature are likely to discover catalysts that can produce greater PHIP than those currently used. Investigation into hydrogenation catalytic cycles focussed on PHIP are likely to stimulate new and improved systems that retain the para-H_2_ spin state more efficiently during catalytic transformations, and give rise to larger NMR signal enhancements for hydrogenated products.

PHIP catalysis performed in coordinating solvents including methanol, ethanol or acetone can give high hydrogenation efficiencies and high NMR signal enhancements.^96,97^ This has been linked to the relatively high para-H_2_ solubility in these solvents, and is also closely related to the role of metal-solvent adducts in catalysis. Displacement of norbornadiene in [Rh(norbornadiene)(dppb)][BF_4_] to form active [Rh(solvent)_2_(diphosphine)][BF_4_] can be favoured in such solvents. Readily-coordinating solvents also favour rapid elimination of hydrogenated products from the metal coordination sphere, which limits the destructive influence of nuclear spin relaxation on PHIP of the products while bound to the metal centre.^96^ Commonly-used PHIP catalysts are typically most soluble in organic solvents (such as methanol, ethanol, acetone, chloroform, dichloromethane, toluene, *etc.*) but exhibit limited solubility in aqueous solutions. Many have sought to increase catalyst solubility in water by modification of spectator ligands (Fig. 4d).^44,98^ For example, [Rh(norbornadiene)(dppb)][BF_4_]-derived complexes in which the phenyl groups of the 1,4-bis(diphenylphosphino)butane ligand are modified to contain OMe and/or SO_3_H groups have been used to perform PHIP of dimethyl maleate in D_2_O.^44^ Related [Rh(norbornadiene)(THP)_2_][BF_4_] catalysts that contain monodentate tris(hydroxymethyl)phosphine (THP) ligands are also water-soluble and have been used to hydrogenate 2-hydroxyethyl 1-^13^C-acrylate-*d*_2,3,3_ yielding hyperpolarised 2-hydroxyethyl 1-^13^C-propionate-*d*_2,3,3_.^98^ Also ionic liquids based on rhodium salts have been used as hydrogenation catalysts to achieve PHIP in aqueous solvents.^73^ Further development of highly active water-soluble PHIP catalysts are likely to facilitate hyperpolarisation of biologically relevant molecules, which may have applications in NMR imaging.^4,6^

The use of cheaper and more readily available metals as catalysts in symmetry-breaking hydrogenation reactions has also been explored (Fig. 4e). Consequently, PHIP has been used to study the hydrogenation of molecules such as phenylacetylene^53^ or diphenylacetylene^47,54^ by palladium phosphine^47,54^ or palladium bisimino^53^ complexes. In the last few years catalysts based on cobalt such as [Co(bis(mesityl-benzimidazol-2-ylidene)phenyl)(pyridine)] have been used to hydrogenate ethyl acrylate with ^13^C signal enhancements of *ca.* 1130-fold at 11.7 T for the terminal site of the hydrogenated product in acetone-*d*_6_.^59^ This is significantly larger than the *ca.* 250-fold achieved under analogous conditions using a traditional [Rh(COD)(dppb)][BF_4_] catalyst.^59^ The scope of PHIP studies will expand as catalysts are improved to optimise their functional group tolerance, performance, speed, and many other factors. We also note that other metals such as V, Zr, Ta, Ce and Mo have been used to demonstrate PHIP with heterogeneous catalysts,^99–101^ and therefore the list of possible metal-based homogeneous catalysts may be extended significantly in the future.

### 5.2 Ruthenium catalysts for *trans* and *geminal* alkene hydrogenations

**Fig. 5:**
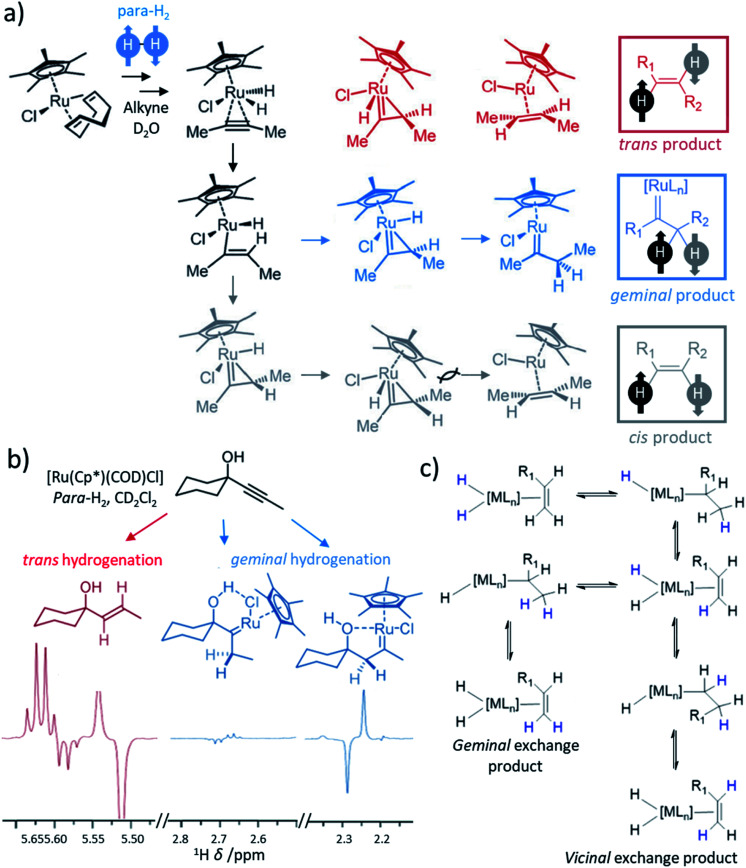
(a) Proposed mechanism for the hydrogenation of alkynes, in this example 2-butyne, by ruthenium cyclopentadienyl complexes yielding products that can be hyperpolarised according to traditional PHIP, *trans*, or *geminal* PHIP. Adapted from ref. 105. Similar mechanisms are given in ref. 106 and 107, although binuclear mechanisms involving two ruthenium centres have also been proposed.^104^ (b) Example partial ^1^H NMR spectra showing enhanced signals following *trans* hydrogenation of the indicated alkyne using a ruthenium catalyst. Enhanced ^1^H NMR signals for ruthenium carbenes result from *geminal* hydrogenation. Adapted from ref. 106. (c) Depiction of metal-catalysed *vicinal* and *geminal* exchange in which proton sites on an alkene are exchanged with a metal dihydride. If a dihydride has formed from activation of para-H_2_, then this exchange can lead to enhanced ^1^H NMR signals for the exchanged sites in the alkene.

Most transition metal-catalysed alkyne hydrogenations involve the formation of *Z* (*cis*) alkenes and is a consequence of concerted hydrogen activation in which the introduced hydride ligands are located in a *cis* relationship.^40,102^ This typically restrains the types of molecules that can be produced from the homogeneous hydrogenation of alkynes in a hyperpolarised state to *Z* alkenes. *E* (*trans*) alkenes can form from homogeneous alkyne hydrogenation, although in many cases this process is actually a *cis* hydrogenation followed by isomerisation from the *Z* to *E* alkene mediated by the metal hydrogenation catalyst.^103^ Such *trans* hydrogenations can occur when heterogeneous solid surface catalysts are employed. In recent years, examples of homogeneous water-soluble ruthenium pentamethylcyclopentadiene (Cp*) catalysts have arisen that are able to *trans*-hydrogenate internal alkynes^104^ in a process that typically involves carbene intermediates (Fig. 5a).^105,106^ These [Ru(Cp*)(L)_*n*_] systems have been reported to react with alkyne and H_2_ to form a [Ru(Cp*)(H)_2_(alkyne)] intermediate that breaks para-H_2_ symmetry by oxidative addition.^105^ Subsequent hydride migration and rearrangement can form a ruthenium carbene intermediate which can react either to give *cis* or *trans* hydrogenated products depending on the geometry of this intermediate. Hydrogenation in a *trans* fashion is driven by steric factors in these intermediates^105^ and can be favoured by parameters such as temperature and solvent.^104,107^ These novel catalysts have expanded the scope of PHIP by allowing the preparation of *E*-alkenes in a hyperpolarised state (Fig. 5b). The formation of *E*-alkenes from alkynes *via* ruthenium carbene intermediates can also produce *geminal* hydrogenated products in which both ^1^H nuclei of the starting para-H_2_ are transferred to a single (rather than adjacent) carbon atom (Fig. 5a).^105,106,108^ While the production of hyperpolarised CH_2_ groups *via geminal* PHIP is possible (Fig. 5b), a challenge of this approach is the slower reaction times compared to traditional PHIP.^109^ Fast relaxation of the newly introduced CH_2_ protons can limit signal enhancements of these sites. Nevertheless, an appropriate resonant radiofrequency field can preserve the singlet spin order within the CH_2_ group which decays with the relaxation time constant for the long-lived state, *T*_LLS_, which can be longer than *T*_1_.^109^ In these systems, *trans* and *geminal* hydrogenations can both occur as competing pathways.^53,109^ Examples of hyperpolarised products resulting from both *trans* and *geminal* PHIP catalysis are expected to increase in the future as more catalytic systems that can perform such chemistry are developed. This has been particularly important for the production of hyperpolarised fumarate which can be used as a metabolic imaging probe and is discussed in more detail in Section 8.1.

### 5.3 Metal-catalysed *vicinal* and *geminal* proton exchange leading to PHIP

Exchange of *vicinal* and *geminal* alkene protons is often a feature of metal-catalysed alkyne or alkene hydrogenation.^53,110–112^ In this process, protons on an alkene can become scrambled *via* metal hydride intermediates. In the context of PHIP experiments, these kinds of metal-catalysed proton exchange events provide a route to introduce parahydrogen-derived protons into alkenes (Fig. 5c). This can occur following a series of hydride migration steps, with bond rotation in the intermediate facilitating the scrambling of these sites. Such reactions can typically be revealed by analogous reaction of D_2_ gas which will exchange with proton sites on the alkene to form H_2_ gas and deuterated alkene.^53^ Similarly, PHIP NMR experiments utilising para-H_2_ can also reveal the occurrence of these reactions from the appearance of hyperpolarised ^1^H NMR spectra. For example, reaction of styrene and para-H_2_ or D_2_ with Rh and Pd catalysts result in scrambling of the para-H_2_ or D_2_ labels with the *geminal* alkene protons.^53^ Similar exchange of para-H_2_ protons with *vicinal* or *geminal* alkene protons has been reported for Rh systems.^112^ Consequently, ^1^H NMR signals for these sites can become enhanced using PHIP. It is important to note that *vicinal* or *geminal* exchange of a single H or D by itself is usually insufficient to give rise to the PHIP effect as both para-H_2_ atoms must be exchanged for the transfer to be pairwise.^53^ The catalysis underlying these NMR signal enhancements does not necessarily require hydrogenation of the alkene, or formation of an alkene from alkyne hydrogenation. Therefore, while *geminal* and *vicinal* exchange often occur in parallel to hydrogenation chemistry, they are reversible and do not necessitate any net changes to the chemical structure of hyperpolarised molecules. Examples of this reactivity in PHIP studies is unusual, likely due to competing hydrogenation that occurs *via* the same metal intermediates. Nevertheless, they provide unforeseen opportunities for hyperpolarisation at CC double-bonds and analysis of hyperpolarised line shapes can give valuable information on these processes, which may often go unidentified.^112^

### 5.4 Homogeneous hydrogenation of CN groups

**Fig. 6:**
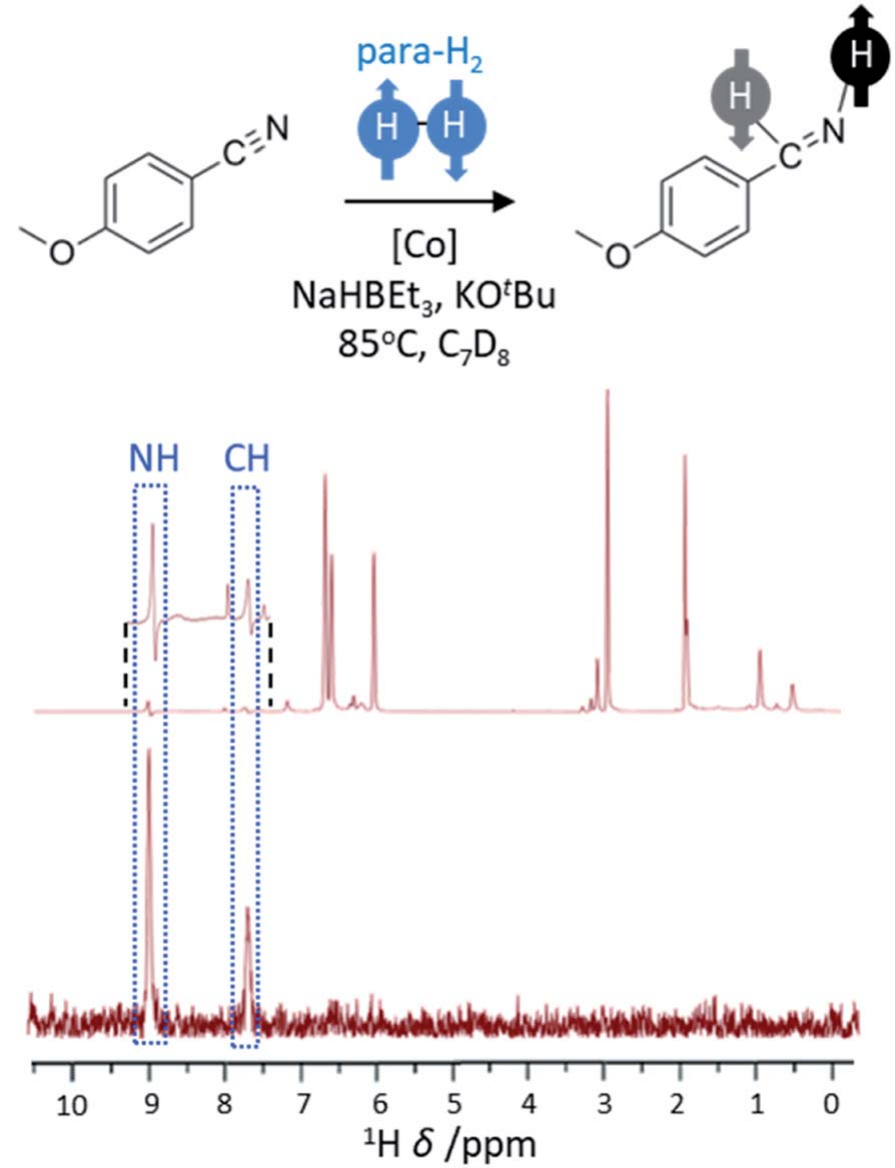
PHIP effects can be observed when nitriles are hydrogenated using para-H_2_. The example ^1^H NMR spectrum (600 MHz) has been recorded for the reaction mixture shown using a 45° pulse (above) with enhanced imine signals (inset) and a ^1^H-OPSY sequence (below) that confirms these protons are derived from para-H_2_. Adapted from ref. 58.

The hydrogenation of unsaturated CC bonds, such as alkynes and alkenes, represents the most common example of substrates used in PHIP studies. However, the hydrogenation of other unsaturated functionalities with para-H_2_ has also been reported, although examples of these are rare. The hydrogenation of nitriles to imines and primary amines using a cobalt bis(carbene) pincer catalyst has been demonstrated (Fig. 6).^58^ This system involves reduction of the cobalt precursor with NaHBEt_3_ before the hydrogenation of CN triple bonds *via* a Co(i/iii) redox process that includes an oxidative addition step.^58^ This process is facilitated by the presence of a Lewis acid (such as BEt_3_) which promotes the η^2^ coordination of the nitrile important for initial transfer of para-H_2_ to form imine. In these examples, hyperpolarised NMR signals for the imine are observed which suggest it is formed from nitrile hydrogenation in a pairwise fashion. However, no hyperpolarised signals for the amine products are observed which is indicative either of a non-pairwise hydrogenation of imine to form amine, or that the pairwise formation of amine is slow compared to the rate of relaxation. These catalysts have been used to hydrogenate a wide range of nitriles^58^ (>20) and indicates that a greater variety of functionalities may become amenable to hyperpolarisation using para-H_2_ as new catalysts to perform these transformations are developed.

### 5.5 Metal-free alkyne hydrogenation

**Fig. 7:**
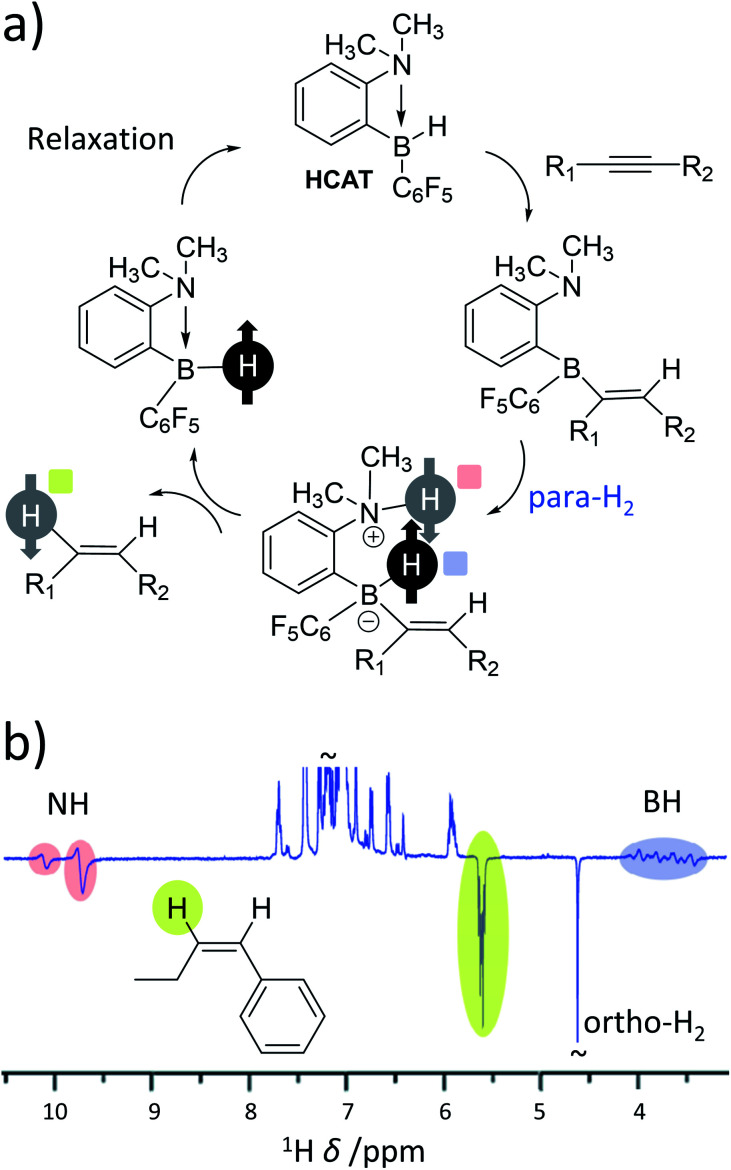
(a) Mechanism of metal-free hydrogenation and PHIP of alkynes using the *ansa*-aminoborane HCAT. Notably, para-H_2_ protons are split heterolytically and only one of these protons is incorporated into the alkene product. (b) Example ^1^H NMR spectrum for an alkene product displaying enhanced ^1^H NMR signals for a single proton originating from the para-H_2_ feedstock in addition to the NH and BH sites of catalytic intermediates. Adapted from ref. 28.

As the range of metal-based systems for PHIP is increasing, so too are examples of metal-free systems. Very recently, *ansa*-aminoboranes have been shown to hydrogenate alkynes with para-H_2_ leading to hyperpolarisation of alkene products.^28^ In the reported examples, a frustrated Lewis pair (HCAT) reacts with an alkyne to form a HCAT-alkyne adduct (Fig. 7a). This adduct performs heterolytic splitting of para-H_2_ as the key step in the catalytic cycle to form a HCAT-alkyne-H_2_ species. Hyperpolarised ^1^H, ^15^N and ^11^B NMR signals for the NH and BH sites are observed which is consistent with similar para-H_2_ activation by FLPs (*vide supra*). However, HCAT-alkyne-H_2_ species can eliminate alkene products with enhanced NMR signals (Fig. 7b). This is the first example of a metal-free catalytic system capable of producing hyperpolarised products other than para-H_2_ activation adducts. Interestingly, only one proton originating from para-H_2_ is transferred into the alkene product, with the other remaining within the HCAT catalyst. This highlights that in some cases both protons originating from the same para-H_2_ molecule need not end up in the same product molecule. Similar effects have been reported for reversible iridium-catalysed exchange of para-H_2_ and H_2_O,^113^ and metal-catalysed hydroformylation reactions (oneH-PHIP as discussed in the next section), although the catalysis and/or polarisation mechanisms involved in these processes are different in each case.

## 6. Hydroformylation reactions

**Fig. 8:**
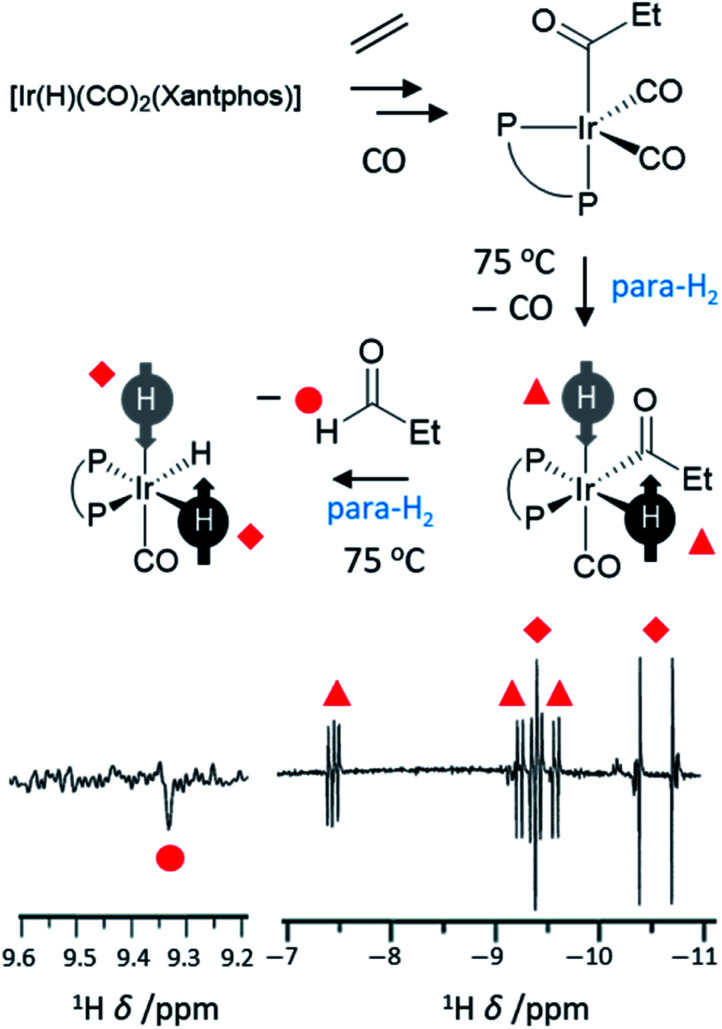
Example oneH-PHIP ^1^H NMR spectrum showing polarisation for propanal (lower left) and for iridium hydride intermediates formed during the hydroformylation process (lower right). Xantphos = 4,5-bis(diphenylphosphino)-9,9-dimethylxanthene adapted from ref. 101.

Hydroformylation reactions, in which alkenes are converted into aldehydes, present a different type of reaction that can break para-H_2_ symmetry leading to PHIP. These reactions lead to the so-called oneH-PHIP effect as only a single ^1^H nucleus originating from para-H_2_ ends up in the aldehyde product but nonetheless can exhibit a net hyperpolarisation.^23^ Despite the fact that the full catalytic cycle in this case is non-pairwise, a pairwise process must occur for PHIP effects to be observed at initial reaction steps. This is achieved by an initial oxidative addition reaction in which both ^1^H nuclei within para-H_2_ are transferred to an intermediary metal dihydride complex, before only one of these hydrides is incorporated into the final product (Fig. 8). One of the first examples of oneH-PHIP was in the hydroformylation of ethene in C_6_D_6_ using [Ir(H)(CO)_2_(dppe)].^23^ Upon reaction of this precursor with 3 bar para-H_2_ at 80 °C, [Ir(COEt)(H)_2_(CO)(dppe)] is formed in which the two ^1^H nuclei from para-H_2_ are strongly coupled and thereby can gain a significant level of single-spin net polarisations of opposite signs. Reductive elimination of this acyl dihydride intermediate yields propanal with an enhanced aldehyde CHO proton due to oneH-PHIP. Hyperpolarised signals for the metal product of reductive elimination, [Ir(H)(CO)(dppe)], were not observed which was attributed to either short relaxation times for this intermediate, or a short lifetime due to rapid formation of [Ir(H)(CO)_2_(dppe)].^23^ Hyperpolarised signals for a [IrH_3_(CO)(dppe)] species are also observed which presumably forms from activation of para-H_2_ by [Ir(H)(CO)(dppe)].^23^

These initial studies also utilised Pt-based systems and since then the range of catalysts used for oneH-PHIP have increased. For example, [Ir(CO)(PPh_3_)_2_(η^3^-C_3_H_5_)]^25,114^ or [IrI(CO)_2_(xantphos)]^24^ hydroformylation catalysts have been employed and consequently, several PHIP-hyperpolarised iridium acyl and alkyl dihydride intermediates could be detected using NMR. The use of less well-defined cobalt systems to perform such oneH-PHIP reactions has also been studied.^26,27^ A wider range of carbonylation reactions, in which CO is incorporated into a substrate, are also accessible to oneH-PHIP. For example, a palladium bis-phosphine complex was demonstrated to convert diphenylacetylene into methyl-2,3-diphenyl acrylate in the presence of CO and H_2_.^55^ This transformation was accompanied by the formation of *cis*- and *trans*-stilbene and the ^1^H NMR signals for all three products were enhanced when this process was performed using para-H_2_. In the case of stilbene, enhanced signals are due to hydrogenative PHIP, although in methyl-2,3-diphenyl acrylate the enhanced ^1^H NMR signals were attributed to a oneH-PHIP effect in which a single proton from para-H_2_ was incorporated into the substrate molecule.^55^

The ^1^H NMR signal enhancements observed for aldehyde protons using oneH-PHIP are significantly lower than can be achieved for alkene and alkanes produced using standard PHIP hydrogenation reactions. Nevertheless, strongly enhanced NMR signals for metal hydride intermediates has been of great use in mapping hydroformylation reaction pathways.^23–27,114^ OneH-PHIP has expanded the types of reactions that can be examined using para-H_2_ and has been of tremendous value in probing inorganic reaction mechanisms.

## 7. Reversible reactions with para-H_2_ and polarisation transfer to other molecules (SABRE)

**Fig. 9:**
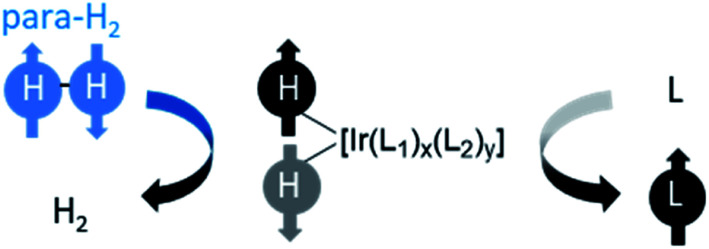
Generic depiction of reversible polarisation transfer using SABRE. The symmetry of para-H_2_ is broken using a reversible oxidative addition reaction to form a hyperpolarised metal dihydride complex. This catalyses spontaneous polarisation transfer to other ligands (L_1_) bound within the complex at low magnetic field (>milliTesla). Auxiliary ligands (L_2_), which can be non-exchanging, often play a role in this process where *x* + *y* = 4. The iridium complex may be neutral or positively charged depending on the identity of L_1_ and L_2_ and is often formed *in situ*.

The reactions discussed so far all result in functionalisation of a to-be-hyperpolarised molecule with protons previously located in the para-H_2_ feedstock. It was first reported in 2009 that target substrates can become hyperpolarised without incorporation of para-H_2_ originating atoms.^29^ The method has been called signal amplification by reversible exchange (SABRE) and it works on the basis of reversible interactions of both para-H_2_ and a to-be-hyperpolarised molecule with a metal catalyst.^16,17,29^ The symmetry of para-H_2_ is initially broken in a reversible oxidative addition reaction to form an iridium dihydride. The difference is that polarisation is then transferred from the hydride ligands to other ^1^H sites or even heteronuclei of ligands bound transiently within the metal complex. Dissociation of the active SABRE catalyst generates hyperpolarised ligand free in solution without hydrogenation of the ligand (Fig. 9). A key advantage of SABRE compared to hydrogenative PHIP is that substrates are not chemically altered. Therefore, SABRE removes the requirement for target molecules to contain unsaturated functionality, although they must now contain motifs that can ligate to the SABRE catalyst. An added benefit is that SABRE is reversible: once the hyperpolarised signals have decayed due to relaxation, they can be regained by shaking or bubbling with fresh para-H_2_. This is in contrast to hydrogenative PHIP which is only visible if the unsaturated substrate is not consumed completely in the reaction.

The spin dynamics of polarisation transfer within SABRE catalysts can be complex and there have been several excellent treatments of these effects.^17,19,115,116^ Briefly, polarisation transfer from the para-H_2_ originating proton pair to target sites is enabled by spin–spin couplings connecting the two hydrides to each other and to the target ligands in the SABRE complex.^17,19,116,117^ Typically, most efficient transfer to ^1^H sites is achieved at 0–10 mT magnetic fields^118,119^ which are often produced in the stray fields of strong NMR magnets (>7 T). It is possible to transfer magnetisation from hydride ligands directly to heteronuclei including ^15^N,^120–124^^13^C,^125–127^^19^F,^128,129^^31^P,^39,130^^119^Sn^131^ and ^31^Si^131,132^ in a target molecule. Heteronuclear SABRE follows the same principles as ^1^H-SABRE but must be performed at much lower magnetic fields (0.5–20 μT) in order to meet resonance conditions.^39,121^ Polarisation transfer within the SABRE catalyst is spontaneous at low magnetic fields (ALTADENA conditions), but it can also occur *via* radiofrequency excitation at high field (Tesla) (PASADENA conditions).^133–138^ In addition, feasible, but less efficient, polarisation transfer can occur spontaneously at high magnetic fields, giving rise to high-field SABRE effects.^133^

### 7.1 Ligand exchange pathways important for SABRE catalysis

**Fig. 10:**
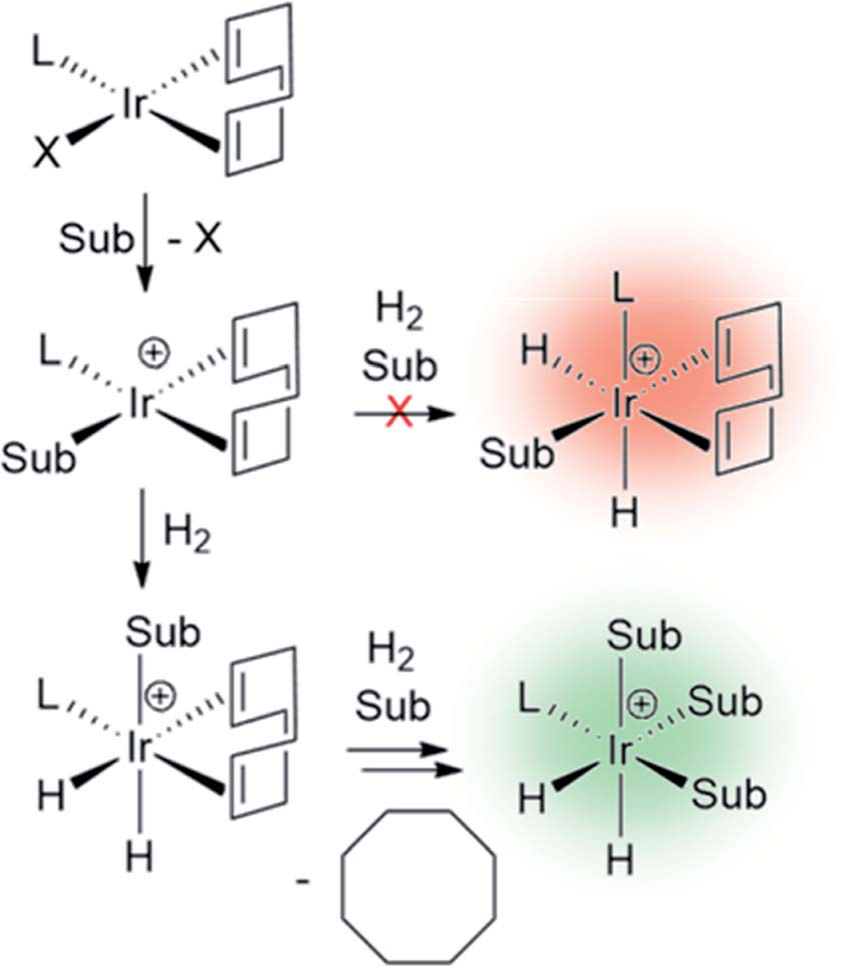
Formation of SABRE-active polarisation transfer catalysts from a 16-electron air-stable precatalyst. In this case L represents an auxiliary ligand and Sub a substrate of interest. The charges of the complexes shown in this scheme will be neutral or positive depending on the identity of X.

The catalysts involved in SABRE are typically of the form [Ir(H)_2_(L)(substrate)_3_]^+^ where L is an auxiliary ligand. Such complexes are formed *in situ* by the reaction of 16-electron square planar precatalysts such as [Ir(COD)(L)(MeCN)][BF_4_] or [IrCl(COD)(L)] (where COD is *cis*,*cis*-1,5-cyclooctadiene) with para-H_2_ and an excess of the substrate of interest.^29^ These reactions typically proceed *via* 16-electron intermediates of the form [Ir(COD)(L)(substrate)]^+^, in which a labile ligand within the precatalyst is replaced by the substrate.^48,139^ Addition of H_2_ to square planar [IrCl(COD)(L)] initially forms the octahedral 18-electron [Ir(H)_2_(COD)(L)(substrate)]^+^. This must undergo hydride migration to form the SABRE-active [Ir(H)_2_(L)(substrate)_3_]^+^ (Fig. 10).

**Fig. 11:**
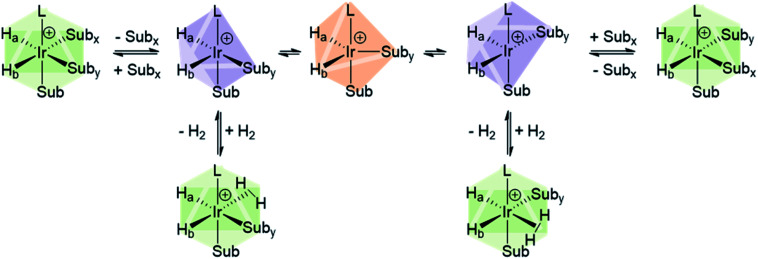
Depiction of isomerisation pathways in SABRE complexes *via* the five coordinate intermediate [Ir(H)_2_(IMes)(Sub)_2_]^+^.

Studies of ligand exchange within these complexes using exchange spectroscopy (EXSY) has revealed that substrate ligands located *trans* to hydride are typically in reversible exchange with their counterpart free in solution.^140^ This reversible exchange allows significant hyperpolarisation for the substrate free in solution to be built up constantly. Mechanistic studies have shown that substrate loss from [Ir(H)_2_(L)(substrate)_3_]Cl to form the 5-coordinate 16-electron intermediate [Ir(H)_2_(L)(substrate)_2_]Cl is a key step in the catalytic SABRE process.^38,141^ The formation of this intermediate (*via* substrate dissociation) is the dominant pathway for both hydrogen and substrate exchange. For example, such transient intermediates can react with para-H_2_ to form [Ir(H)_2_(η^2^-para-H_2_)(L)(substrate)_2_]Cl which undergoes rapid rearrangement to eliminate H_2_ (Fig. 11). The 5-coordinate [Ir(H)_2_(L)(substrate)_2_]Cl exists in a square-based pyramidal geometry immediately after substrate dissociation.^141^ However, formation of a trigonal bipyramidal structure is possible if the complex has a sufficiently long lifetime for ligand reorientation. Isomerisation between different square-based pyramidal structures *via* a trigonal bipyramidal form, or reaction of a trigonal bipyramidal intermediate with either para-H_2_ or substrate on either face, is responsible for scrambling of the hydride ligand sites (Fig. 11).^142^ This becomes more important for complexes that contain inequivalent hydride ligands.^38^

For catalytic polarisation transfer to be observed, [Ir(H)_2_(η^2^-para-H_2_)(L)(substrate)_2_]Cl must be short-lived to limit the conversion of para-H_2_ into ortho-H_2_.^143^ Instead, [Ir(H)_2_(η^2^-para-H_2_)(L)(substrate)_2_]Cl serves to refresh the para-H_2_-derived singlet spin order within the iridium dihydride catalyst. The rate of H_2_ loss typically decreases as the substrate concentration is increased, but increases as hydrogen pressure is increased.^38,48,141^ These observations have been used to deduce that hydrogen and substrate exchange occur through a common pathway in which both hydrogen and substrate bind competitively to intermediates such as [Ir(H)_2_(L)(substrate)_2_]Cl. In other words, pathways involving hydrogen loss directly from [Ir(H)_2_(L)(pyridine)_3_]Cl to form [Ir(L)(pyridine)_3_]Cl with changes in oxidation state are unlikely.^38,141^ The transient intermediates involved in SABRE catalysis are often too short-lived to be observed directly by NMR methods, although Density Functional Theory (DFT) calculations have provided supporting evidence for these mechanistic studies.^38,141,144^ These studies have confirmed that direct reductive elimination of hydrogen is accompanied with a large energy barrier, favouring a pathway involving species with constant oxidation states.

### 7.2 Variation of the auxiliary ligand to fine tune substrate exchange and relaxation

The SABRE catalyst plays a crucial role in determining the substrates that can become hyperpolarised, and the magnitude of the NMR signal enhancements that can be achieved. The lifetime of the active SABRE catalyst is of vital importance: if it is too short then ligand exchange will occur before there has been sufficient time for a substrate to become hyperpolarised, if it is too long then the hyperpolarisation of substrate will decay due to the enhanced spin relaxation while bound to the metal centre. The active catalyst lifetime is closely linked to the substrate exchange rate, which can be altered by variation of auxiliary ligands on the catalyst, although factors such as temperature^122,145,146^ or even solvent will also influence this.^142,147^ The steric and electronic properties of the auxiliary ligand can also influence the types of substrates that can bind to the SABRE catalyst. Therefore, careful catalyst optimisation can play a significant role in tuning ligand exchange which in turn provides a valuable route to improving the substrate scope and hyperpolarisation levels achieved using SABRE. Novel pulse sequences have also been used as a route to achieve high polarisation by compensating for suboptimal ligand exchange processes.^148^

Catalysts involved in early SABRE studies contained an auxiliary phosphine ligand and were of the form [Ir(H)_2_(PCy_3_)(substrate)_3_][BF_4_], where Cy is cyclohexyl. It was soon noted that the identity of the auxiliary phosphine ligand was one of many important factors that determine the efficiency of SABRE catalysis and the resulting NMR signal enhancements that can be achieved. For example, catalysts containing the phosphine ligand PCy_2_Ph yielded higher ^1^H NMR enhancements for pyridine compared to the use of catalysts derived from other phosphines including Pcy_3_ and PPh_3_.^149^ Sterically large electron-rich phosphines are preferred which is attributed to more favourable substrate dissociation.

**Fig. 12:**
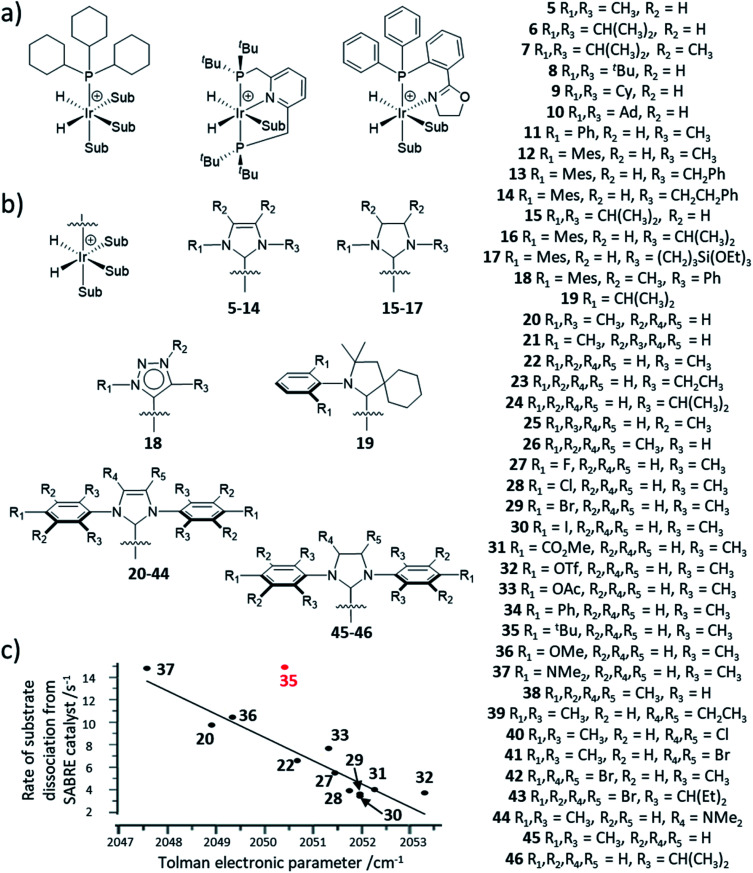
(a) Example iridium phosphine complexes used for catalytic polarisation transfer from para-H_2_. Reported examples are based on traditional monodentate κ^1^-*P* phosphine ligands^11,150,151^ (left), κ^3^-*N*,*P*,*P* pincer ligands^154^ (middle) or κ^2^-*N*,*P* phox ligands^156^ (right). (b) SABRE catalysts containing different auxiliary NHC ligands. (c) Effect of *para* substituted NHC ligands as a function of their TEP on the rate of substrate dissociation from [Ir(H)_2_(NHC)(sub)_3_]Cl at 298 K where substrate is methyl-4,6-*d*_2_-nicotinate. The outlier, 35 (shown in red), is attributed to steric effects of the bulky ^*t*^Bu group. Adapted from ref. 153.

Since then, a variety of more complex phosphine ligands have been used that contain mixed P and N donor sites. Namely, iridium pincer complexes containing a κ^3^-*N*,*P*,*P* auxiliary ligand have been used as polarisation transfer catalysts (Fig. 12a).^142^ Related iridium κ^2^-*N*,*P* systems that use a 2-(2-(diphenylphosphanyl)phenyl)-4,5-dihydrooxazole (Phox) ligand have also been reported.^150^ This catalyst achieved much lower SABRE efficiency for pyridine (2-fold *ortho*^1^H NMR signal enhancement) compared to using [Ir(H)_2_(PCy_3_)(pyridine)_3_]PF_4_ (100-fold *ortho*^1^H NMR signal enhancement).^150^ However, the significant advantage of these Phox-containing systems is that they are able to hyperpolarise sterically larger substrates. In particular, pyridines with substituents at the *ortho* position such as 2-methylpyridine, 2-fluoropyridine, and 2-ethylpyridine exhibit ^1^H NMR signal enhancements for their aromatic *ortho* sites of 132-, 140-, and 25-fold respectively^150^ and could not be hyperpolarised using [Ir(H)_2_(PCy_3_)(substrate)_3_]PF_4_.

It was soon discovered that the use of carbene ligands, instead of phosphines, has a large effect in increasing the efficiency of SABRE catalysis. Their greater SABRE efficiency has been rationalised in terms of optimised substrate exchange within the active catalytic cycle. The rate constant of pyridine dissociation from [Ir(H)_2_(IMes)(pyridine)_3_]Cl was determined by EXSY to be 23 s^−1^ at 300 K (ref. 48) which is *ca.* > 50 times faster than pyridine dissociation from [Ir(H)_2_(Pcy_3_)(pyridine)_3_]^+^ (0.45 s^−1^ at 295 K).^149^ As a consequence, ^1^H NMR signal enhancements of 8100-fold could be achieved for pyridine in methanol-*d*_4_ at 3 T using an [Ir(H)_2_(IMes)(pyridine)_3_]Cl active catalyst^141^ which is much higher than those achieved using phosphine-based catalysts (<100-fold at 9.4 T).^149^

**Fig. 13:**
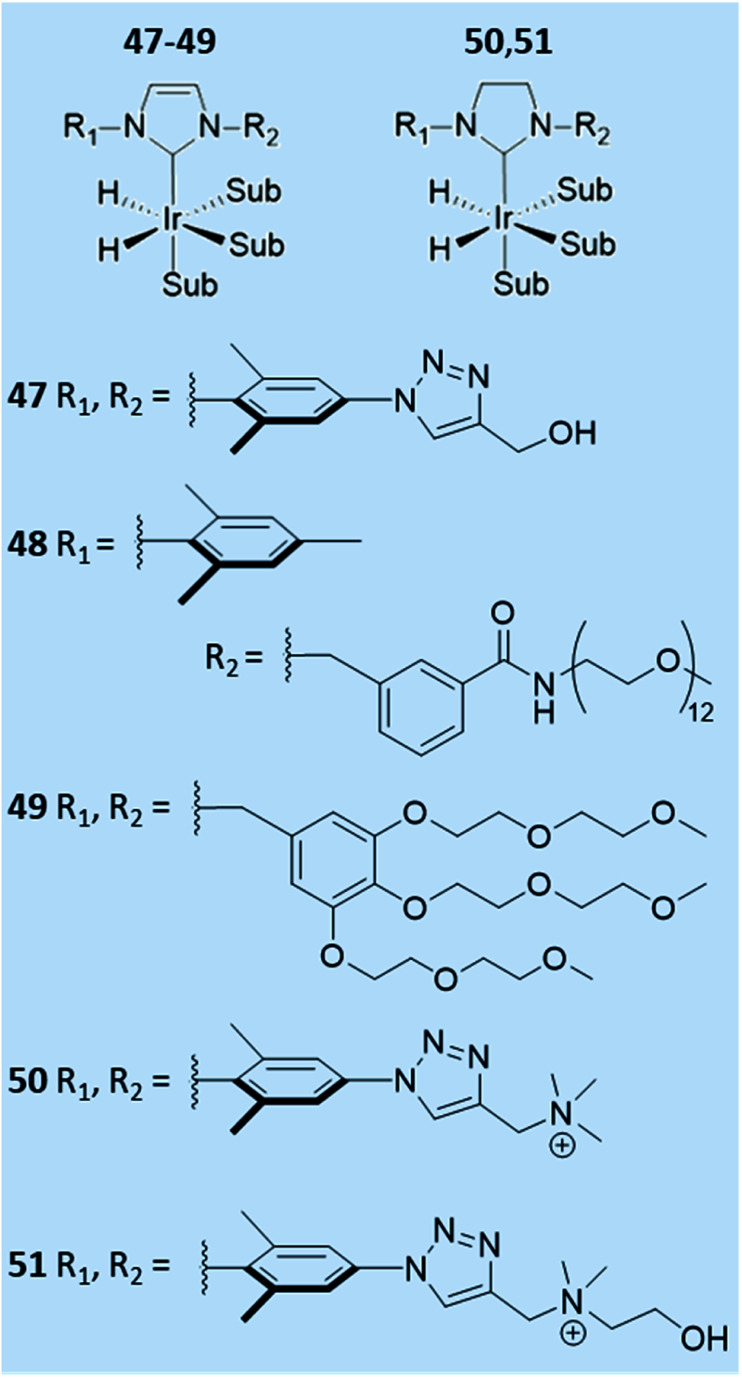
Reported examples of water-soluble SABRE catalysts.

The NHC ligand used to make [IrCl(COD)(NHC)] SABRE precatalysts can easily be modified to exhibit a variety of steric, and to a lesser extent, electronic properties.^151,152^ Consequently, a large variety of SABRE catalysts with different NHC auxiliary ligands have been reported (Fig. 13b, also see ESI Table S1†) and their effect on SABRE efficiency has been investigated.^48,153–156^ Generally, it is those catalysts containing an NHC ligand based on IMes that provide some of the highest NMR signal gains for substrates of interest using SABRE. When comparing IMes-derivatives with variation in the substituent at the para position, R_1_ (20–44), there is a good correlation between electron-rich carbenes (low TEP) and fast rates of substrate dissociation from [Ir(H)_2_NHC(substrate)_3_]Cl (Fig. 12c).^153^ This is generally beneficial for refreshing para-H_2_ spin order and facilitating catalytic build-up of substrate polarisation, although rationalisation of catalyst efficiencies can be challenging. Relaxation within the active catalyst is also an important factor that determines the magnitude of hyperpolarised NMR signals that SABRE can produce. Therefore, selected ^1^H sites within the NHC ligand can be replaced with deuterium (^2^H) labels.^153^ This reduces any wastage of para-H_2_ derived spin order by preventing unwanted transfer to ^1^H sites of the auxiliary ligand. It also serves to reduce relaxation of the substrate when bound to the iridium catalyst and therefore substrate relaxation times in the presence of the catalyst are often longer when deuterium-labelled catalysts are used compared to their protium counterparts.^153^

### 7.3 Variation of the auxiliary ligand for water-soluble catalysts

The effect of the auxiliary ligand can also extend beyond subtle steric, electronic, and relaxation effects. For example, catalyst solubility can be altered by changing the auxiliary ligand. Ir–NHC catalysts are typically highly soluble in organic solvents and therefore polarisation transfer reactions are typically performed in methanol-*d*_4_ (ref. 29, 38, 146, 157 and 158) or even dichloromethane-*d*_2_,^37,38^ chloroform-*d*^159^ or ethanol-*d*_6_.^146^ The efficiency of SABRE catalysis in other solvents can be improved by alteration of the auxiliary NHC ligand. For example, a neutral iridium carbene complex where the NHC was 3-(2-methylene-4-nitrophenolate)-1-(2,4,6-trimethylphenyl)imidazolylidene, which contained a pendant alkoxide ligand, has been utilised in low polarity solvents including benzene-*d*_6_, and THF-*d*_8_.^160^

Water-soluble SABRE catalysts allow the entire hyperpolarisation process to be performed in aqueous solvents. Specifically, auxiliary PPh_3_ ligands were functionalised with SO_2_Na or SO_3_K groups to produce water-soluble catalysts.^161^ A variety of water-soluble IMes-derived ligands have also been developed (Fig. 13).^161–163^ Catalyst 51 was able to efficiently catalyse SABRE polarisation transfer to pyridine in methanol-*d*_4_ giving total ^1^H NMR signal enhancements of 750-fold.^161^ However, when this was repeated in 70 : 30 D_2_O : ethanol-*d*_6_ this value dropped to just 22-fold. Even though 47, 50–51 were soluble in water they failed to yield appreciable NMR signal enhancements for pyridine in water which was attributed to low para-H_2_ solubility.^161^ In contrast, 48 could achieve ^1^H NMR signal enhancements of *ca.* 30-fold for pyridine in water.^162^ Similar signal enhancements were observed for pyridine using 49, with up to 42-fold ^1^H NMR signal enhancement for the *ortho* site of nicotinamide also reported.^163^ These studies have shown that variation of the auxiliary ligand can alter catalyst solubility and they have been successful in allowing reversible polarisation transfer reactions to be performed directly in aqueous solvent. Nevertheless, they have not yet been reported to achieve NMR signal enhancements comparable to those that can be attained in organic solvents. Further alterations of the auxiliary ligand may yield higher-performing water-soluble catalysts that can give more significant NMR signal enhancements in the future.

### 7.4 Coligand-supported SABRE catalysts to hyperpolarise sterically large or weakly donating substrates

For bulky substrates, formation of [Ir(H)_2_(IMes)(substrate)_3_]Cl may be prevented by steric effects.^118,150,158^ The use of catalysts with sterically smaller auxiliary ligands, such as phosphines,^150^ asymmetric carbenes,^164^ or bidentate carbenes^165^ can be used to form SABRE catalysts with bulkier substrate ligands. A more effective way to create stable SABRE catalysts in cases where [Ir(H)_2_(NHC)(substrate)_3_]Cl is not formed due to sterically large, or weakly-ligating, substrates is the use of coligand-supported catalysts.^158,166^ For example, coordination of pyridine-derived substrates with functionality in the *ortho* position that hinders iridium ligation can be facilitated by a coligand.^150,166^ To demonstrate, ^1^H NMR signal enhancements up to 1442 ± 84-fold were recorded for 2,5-lutidine using catalysts of the form [IrCl(H)_2_(NHC)(sulfoxide)(substrate)]. This approach is notable as substrates of this type are not amenable to SABRE using [Ir(H)_2_(IMes)(substrate)_3_]Cl due to steric constraints. These catalysts could also improve upon NMR signal enhancements for molecules such as 2-picoline compared to those achieved using typical [Ir(H)_2_(IMes)(substrate)_3_]Cl systems.^166^ The inclusion of a coligand can in some cases have a beneficial effect on increasing substrate signals enhancements for systems in which [Ir(H)_2_(NHC)(substrate)_3_]Cl is readily formed.^123,167–170^ This is possible if relaxation or ligand exchange is more favourable for [Ir(H)_2_(IMes)(substrate)_2_(coligand)]Cl compared to [Ir(H)_2_(IMes)(substrate)_3_]Cl.

**Fig. 14:**
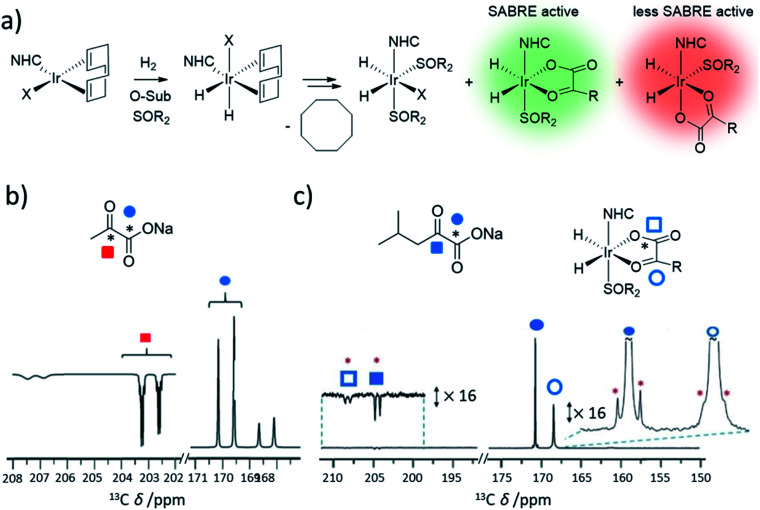
(a) Formation of sulfoxide-containing SABRE catalysts that can hyperpolarise *O*-donor ketoacids. (b) Example use of these catalysts to achieve hyperpolarisation of pyruvate. Partial hyperpolarised ^13^C NMR spectra for the keto region (left) and carbonyl region (right) recorded after [IrCl(COD)(IMes)] (5 mM), methylphenylsulfoxide (50 mM), and sodium pyruvate-1,2-[^13^C_2_] (30 mM) are shaken in methanol-*d*_4_ (0.6 mL) with 3 bar para-H_2_ for 30 seconds in a mu-metal shield. (c) Example use of these catalysts to achieve hyperpolarisation of ketoisocaproate. Partial hyperpolarised ^13^C NMR spectra when [IrCl(COD)(IMes)] (5 mM) and ketoisocaproate-1-[^13^C] are shaken with dimethylsulfoxide (50 mM) and para-H_2_ (3 bar) in methanol-*d*_4_ (0.6 mL) for 10 seconds in a mu-metal shield at *ca.* 1 μT. The signals marked by a red asterisk denote singlet magnetisation of naturally abundant ketoisocaproate-1,2-[^13^C_2_]. Adapted from (b) ref. 146 and (c) ref. 157.

Careful selection of coligands around the metal centre can create SABRE catalysts in which weakly-ligating substrates can bind and become hyperpolarised. Particularly, *O*-donor molecules such as carboxylic acids and ketoacids do not form the [Ir(H)_2_(NHC)(substrate)_3_]Cl typically necessary for polarisation transfer in reversible reactions. However, novel SABRE catalysts containing stabilising coligands have been developed to catalyse polarisation transfer to *O*-donor molecules. For example, ^13^C NMR enhancements of 108-fold for ^13^C-acetate were achieved using [Ir(H)_2_(IMes)(acetate)(pyridine)_2_].^126^ More notably, sulfoxides have emerged as a versatile class of SABRE coligand^38,125,146,147,157^ and as a result, novel SABRE catalysts of the form [Ir(H)_2_(κ^2^-*O*,*O*-pyruvate)(NHC)(sulfoxide)]Cl or [Ir(H)_2_(κ^2^-*O*,*O*-ketoisokaproate)(NHC)(sulfoxide)]Cl can deliver up to 2135 and 985-fold ^13^C NMR signal enhancements for sodium pyruvate-1,2-[^13^C_2_]^146^ and sodium ketoisocaproate-1-[^13^C]^157^ in methanol-*d*_4_, respectively (Fig. 14b and c). Since these initial studies, the efficiency of these catalysts have been improved further by variation of temperature to optimise ligand exchange processes and *ca.* 11% ^13^C polarisation for sodium pyruvate-1-[^13^C] has been reported.^171^

The mechanism of formation, and action, of these sulfoxide-containing catalysts contains several key differences from that of traditional [Ir(H)_2_(NHC)(substrate)_3_]Cl-based systems. There is no evidence of displacement of chloride from [IrCl(COD)(NHC)] by either *O*-donor substrate or sulfoxide.^147^ As a consequence, hydrogen addition occurs directly to [IrCl(COD)(NHC)] to form [IrCl(H)_2_(COD)(NHC)] (Fig. 14).^147,172^ Hydrogenation of the COD ligand then occurs to form [IrCl(H)_2_(sulfoxide)_2_(NHC)] which exists in equilibrium with [Ir(H)_2_(κ^2^-*O*,*O*-substrate)(NHC)(sulfoxide)].^125^ Unusually, [Ir(H)_2_(κ^2^-*O*,*O*-substrate)(NHC)(sulfoxide)] exhibits relatively slow ligand exchange on the NMR timescale.^147^ There are other species also present in solution and those of the form [IrCl(H)_2_(sulfoxide)_2_(NHC)] are known to undergo fast para-H_2_ exchange.^147^ They are therefore likely to play a role in refreshing para-H_2_ spin order within the catalytic system. The role of these species is also highlighted by the fact that the identity of the ligand, X, in [IrX(H)_2_(sulfoxide)_2_(NHC)] can have a large effect on the substrate NMR signals enhancements of *O*-donor substrates.^147^ Catalysis can be further complicated in these examples by the existence of two isomers of [Ir(H)_2_(κ^2^-*O*,*O*-substrate)(NHC)(sulfoxide)].^125,146,171^ It is those species containing substrate ligated in the same plane as the hydride ligands that have a spin topology most appropriate for efficient polarisation transfer to substrate.^125^ The proportion of active isomer can be maximised by alteration of the steric properties of the NHC.^146^ Such systems have allowed the polarisation of previously inaccessible molecules: continued development of novel classes of coligands with unique reactivity will unlock the ability to hyperpolarise an ever-greater range of molecules using SABRE.

### 7.5 Role of low concentration adducts on SABRE catalysis

Some of the most efficient SABRE catalysts have been highlighted. It is worth noting that there are many reports of low concentration adducts formed in solution that are not efficient SABRE catalysts and their presence can often have a detrimental effect on SABRE efficiency.^35,173^ SABRE catalysis is most commonly performed in methanol-*d*_4_, which can act as a coordinating solvent. Therefore, the trapping of short-lived 5-coordinate reaction intermediates, such as [Ir(H)_2_(NHC)(substrate)_2_]Cl, with methanol to form [Ir(H)_2_(NHC)(substrate)_2_(CD_3_OD)]Cl^48,173^ can occur. These adducts are typically of low concentration and are short-lived due to the weak binding of solvent methanol. The role that these adducts play can be revealed by recording SABRE efficiency as a function of polarisation transfer field (PTF).^173^ These PTF plots can include multiple maxima and this has been rationalised in terms of different optimal magnetic fields for polarisation transfer to substrate catalysed by [Ir(H)_2_(NHC)(substrate)_2_(CD_3_OD)]Cl compared to [Ir(H)_2_(NHC)(substrate)_3_]Cl.^173^ These differences are a result of the different *J* coupling spin topology formed in the solvent adduct compared to the traditional tris-substrate catalyst. These studies have shown that the role of undiscerned species in SABRE catalysis can be challenging to determine and may be important for those seeking to model polarisation transfer during SABRE.^19,115,116,174,175^

Other adducts such as [IrCl(H)_2_(NHC)(substrate)_2_]Cl have been reported^35,173^ and they are typically detected *via* distinctive hydride resonances or indirect methods based on CEST.^35^ [IrCl(H)_2_(NHC)(substrate)_2_] has been reported to have a detrimental effect on SABRE efficiency as it results in a loss of para-H_2_ spin order. This loss of correlation, or singlet–triplet leakage, is typically larger at higher magnetic field and it can be reduced by performing SABRE experiments at lower fields^176^ or by applying a spin lock pulse during para-H_2_ bubbling to increase the lifetime of para-H_2_ spin order.^35^

### 7.6 Inefficient Ir–NHC SABRE catalysts: multinuclear decomposition products

**Fig. 15:**
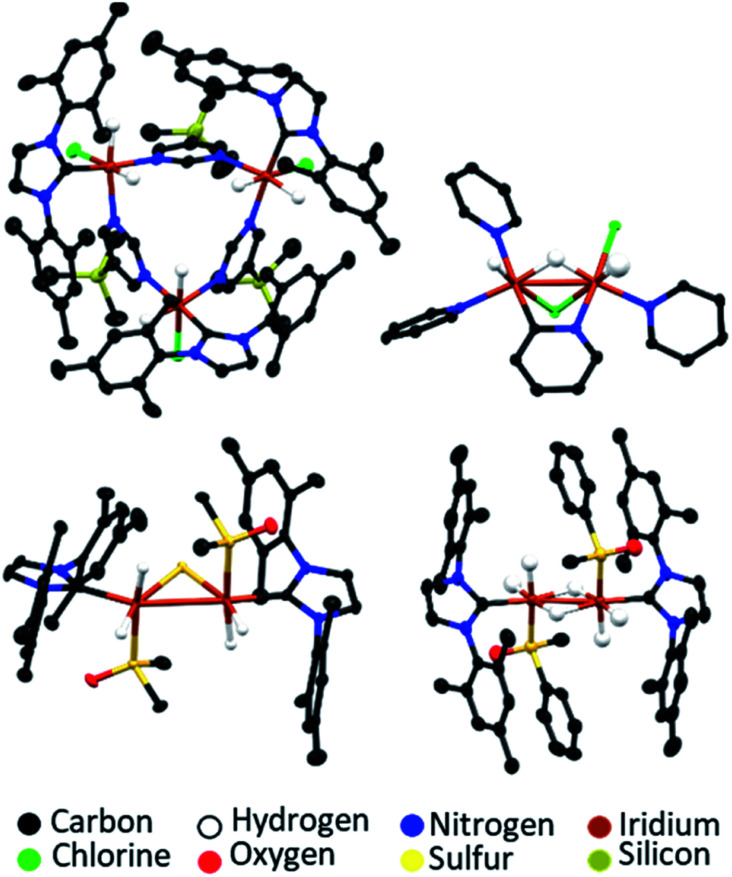
Example X-ray crystal structures of catalyst decomposition products reported in ref. 131 (upper left), ref. 159 (upper right) and ref. 157 (lower). Note that thermal ellipsoids are shown at 50% probability and all non-hydride hydrogen atoms and solvent of crystallisation have been omitted for clarity.

The SABRE-active catalysts discussed so far are all formed over differing activation time periods which can range from several minutes^146^ to several days.^160^ While SABRE can easily be repeated multiple times by adding fresh para-H_2_ and repeating the polarisation step, this cannot occur indefinitely and, as with all catalytic processes, there is catalyst degradation over longer timescales.^131,157^ Different systems will remain active for different time windows, which are usually sufficient to allow many repeated hyperpolarisation measurements to take place. For some catalysts, activity is observed for several hours after catalyst formation. For others, deactivation can be more rapid.^146^ Formation of catalyst decomposition products, which are usually dimers or higher order oligomers, can be responsible for a drop in SABRE efficiency at longer reaction times.^146,177^ The formation of a wide range of dimer or trimer products, which can be revealed using X-ray crystallography, have been reported (Fig. 15).^131,147,157,159,172,177^ These often contain iridium–iridium single bonds^146,157,159,172^ and may also contain bridging hydride,^157,159,172^ chloride,^159^ substrate,^131,147^ or in the case of sulfoxide-containing catalysts, bridging S^2−^ or SR^−^ ligands.^146,157,172^ These species are typically no longer able to undergo the ligand exchange processes necessary for reversible polarisation transfer and therefore do not act as SABRE catalysts.^177^ Hence, their formation should be minimised by modification of the substrate, auxiliary ligand or coligand (if applicable).^146^

### 7.7 Novel catalysts based on cobalt for SABRE-like hyperpolarisation

**Fig. 16:**
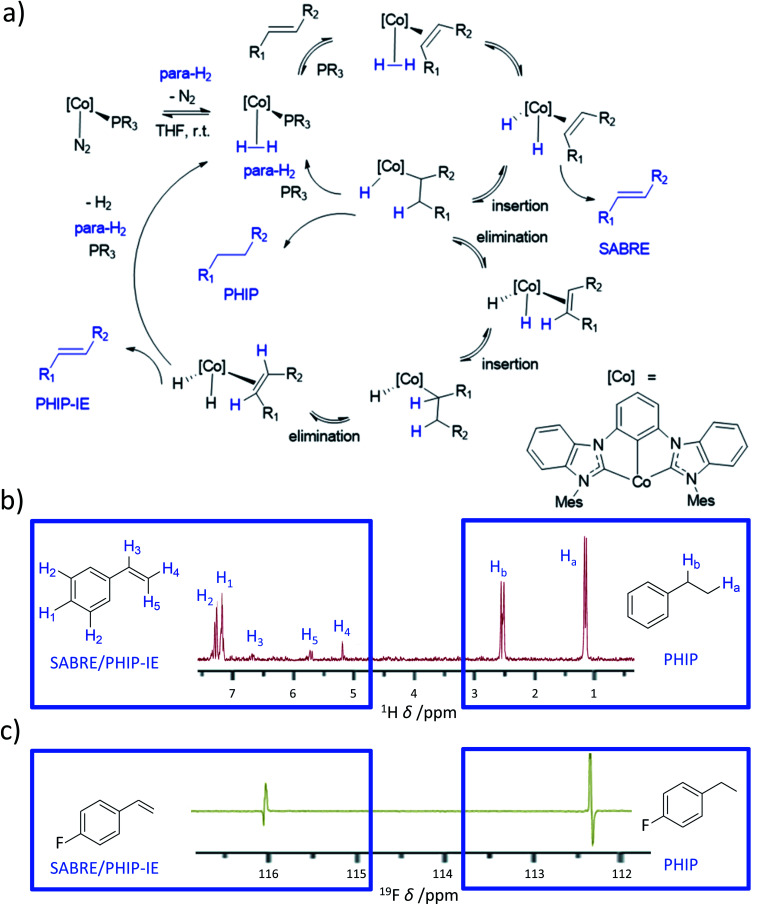
Cobalt-based system used to achieve both PHIP and SABRE-like hyperpolarisation. (a) Simplified mechanism showing ways in which alkenes can become hydrogenated using para-H_2_ to give PHIP for alkane products, or the starting alkene can become hyperpolarised in a SABRE or PHIP-IE pathway. (b) Example ^1^H OPSY spectrum showing enhanced ^1^H NMR signals due to both PHIP and SABRE/PHIP-IE for phenylethane and phenylpropene respectively. (c) ^19^F NMR Spectrum for fluorophenylpropene and fluorophenylpropane hyperpolarised using these cobalt systems. Adapted from (b) ref. 179 and (c) ref. 57.

Almost all currently reported SABRE catalysts are based on iridium. In the future, completely new systems that are not based on Ir-phosphine or Ir–NHC catalysts are likely to become available that may be more efficient than the current state-of-the-art catalysts. This includes discovery of catalysts that are based on other transition metals. Rhodium complexes have been the focus of attempts to create non-Ir SABRE catalysts^178^ as it also resides in group 9 of the periodic table, exhibits similar chemistry to iridium, and is used for hydrogenative PHIP. Despite this, no significant breakthroughs in the development of such Rh-based systems have yet been reported. However, in recent years a novel cobalt dihydride system containing a pincer bis(carbene) ligand has been developed that is capable of hydrogenating alkenes.^59,103,179^ When these reactions were studied using PHIP, NMR signal enhancements for the hydrogenated products were observed, as is typical of the PHIP effect. However, enhanced signals for the alkene reagents were also visible which were attributed to a reversible SABRE-like effect. This is possible due to reaction of [Co(^Mes^CCC)(N_2_)(PR_3_)] (where ^Mes^CCC = bis(mesityl-benzimidazol-2-ylidene)phenyl) with para-H_2_ to form [Co(^Mes^CCC)(H_2_)(PR_3_)] (Fig. 16).^179^ Subsequent phosphine displacement by reaction with an alkene yields [Co(^Mes^CCC)(H_2_)(alkene)]. It is this species that performs a symmetry-breaking oxidative addition to form [Co(^Mes^CCC)(H)_2_(alkene)]. It is worth noting that in these examples [Co(^Mes^CCC)(H_2_)(PR_3_)] does not appear able to undergo the analogous reaction (*i.e.* [Co(^Mes^CCC)(H)_2_(PR_3_)] does not form). The active species [Co(^Mes^CCC)(H)_2_(alkene)] can hydrogenate the alkene to form an alkane, which exists in a hyperpolarised state due to PHIP. As alkene binding must be reversible, it can dissociate chemically unchanged. It also exists in a non-Boltzmann spin state which is a consequence of SABRE-like polarisation transfer within [Co(^Mes^CCC)(H)_2_(alkene)] (Fig. 16).^179^ Recently, these effects have been extended to a wider range of substrates (>15) including styrenes, terpenes, acrylates, and pentenoates with ^1^H and ^13^C signals enhanced by up to 150-fold.^57^ Other mechanisms for these observations, such as insertion–elimination (PHIP-IE) of the alkene to effectively exchange its protons with those derived from para-H_2_, have also been proposed (Fig. 16).^57^ In these cobalt systems, substrates can undergo competing PHIP or PHIP-IE/SABRE^103^ and additional studies are anticipated to further understand the different polarisation transfer mechanisms in action in these systems. Nevertheless, these ongoing studies have utilised a more widely available, cheaper, transition metal (compared to iridium or rhodium), and have demonstrated that reversible polarisation transfer is likely not exclusive to iridium catalysts. It is also expected that these breakthroughs will stimulate further design of novel systems that may exhibit vastly different catalysis to currently used systems.

## 8. Applications of novel PHIP catalysts in hyperpolarisation

In this final section we highlight just two recent applications of PHIP hyperpolarisation in NMR that would not have been possible without the development of novel catalysts that can break para-H_2_ symmetry.

### 8.1 Water-soluble ruthenium catalysts for *trans* hydrogenation: *in vivo* molecular imaging of hyperpolarised fumarate

**Fig. 17:**
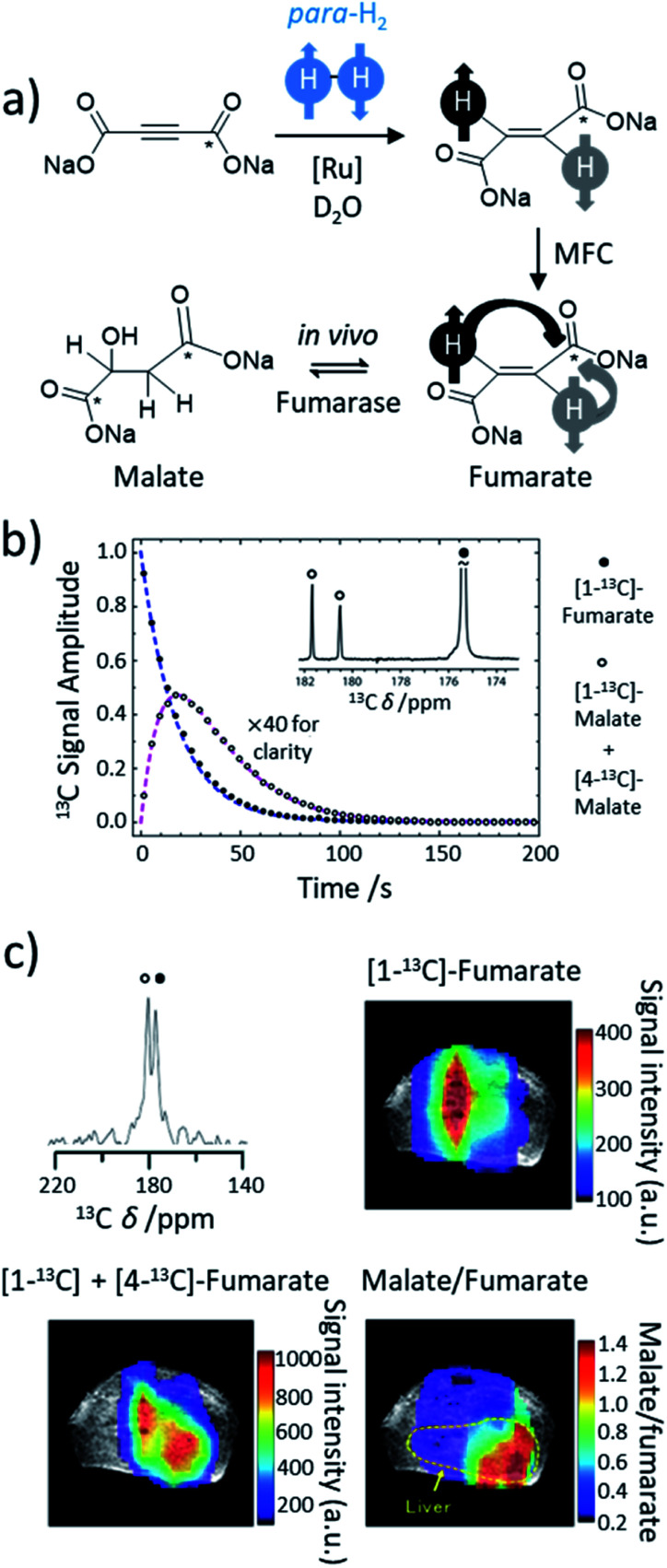
(a) Reaction scheme showing the formation of PHIP-hyperpolarised [1-^13^C]-fumarate from *trans* hydrogenation of an unsaturated [1-^13^C]acetylene dicarboxylate precursor. Proton magnetisation is transferred to the ^13^C site by magnetic field cycling (MFC). Metabolic conversion following *in vivo* injection can give rise to [1-^13^C]-malate and [4-^13^C]-malate products. Note that * represents a ^13^C labelled site and the nuclei represented in black circles are hyperpolarised. (b) Time course of hyperpolarised fumarate and malate in a suspension of lysed EL-4 tumour at 14.1 T with an example single acquisition (inset). Adapted from ref. 182. (c) *In vivo*^13^C chemical shift imaging of PHIP-hyperpolarised [1-^13^C]-fumarate and its metabolic products in an acetaminophen-induced hepatitis mouse at 1.5 T. An example single voxel ^13^C NMR spectrum is shown in the upper left. Maps of hyperpolarised ^13^C signal intensity for [1-^13^C]-fumarate (upper right) and both [1-^13^C] and [4-^13^C]malate (lower left) are shown with a parametric map of the malate/fumarate ratio (lower right). Adapted from ref. 107.

The NMR signal gains that can be provided by hyperpolarisation have provided exciting opportunities for biomolecular imaging.^4,6,180^ MRI is currently used for routine diagnosis of tissue structure abnormalities, but its low sensitivity necessitates detecting a signal for bulk water at tens of molar concentrations in the body. Boosting MRI signal intensity allows molecules at much lower (*i.e.*, millimolar) concentrations such as disease markers, drugs, and many other biologically relevant molecules, to be detected *in vivo*. This dramatically improves the diagnostic value of MRI as it can now give information about tissue biochemistry that are not available in thermally polarised MRI measurements. There are many key metabolic intermediates that are the focus of hyperpolarisation studies as their injection and imaging allows specific metabolic processes to be interrogated *in vivo* in real time.^9^ One of these molecules is fumarate, a key intermediate of the Krebs cycle. Hyperpolarised [1,4-^13^C_2_]-fumarate can be used to probe necrotic cell death by observation of enhanced signals for [1-^13^C] and [4-^13^C]-malate products (Fig. 17).^107,181^ The advent of water-soluble ruthenium catalysts that can perform *trans* hydrogenation reactions unlock the potential for PHIP to produce fumarate in a hyperpolarised state (Fig. 17a).^181,182^ Transfer of polarisation from introduced protons to a ^13^C-labelled site can occur using a magnetic field cycling (MFC) approach and was reported to yield ^13^C signals for [1,4-^13^C_2_]-fumarate with a polarisation level of up to 24%.^182^ It has since been reported that this has been increased to 30–45% by optimisation of the equipment, reaction conditions, and purification procedure (precipitation as a solid before redissolving).^183,184^ The great benefit of this approach is that it is highly compatible with biological imaging studies as it can produce aqueous solutions of hyperpolarised fumarate without contaminant side products or toxic catalyst.^107,183^ Production and injection of PHIP-hyperpolarised [1-^13^C]-fumarate into mice containing acetaminophen-induced hepatitis has allowed fumarate biochemistry to be examined *in vivo* using 2D chemical shift imaging at 1.5 T (Fig. 17c).^107^ This feat would not be possible without development of catalytic systems that can hydrogenate starting unsaturated precursors in a *trans* fashion. Notably, ^13^C polarisation levels for fumarate are comparable to those achieved using alternative hyperpolarisation techniques such as dissolution dynamic nuclear polarisation (d-DNP).^185,186^ However, PHIP provides several advantages compared to d-DNP as it is rapid (seconds-minutes) and does not require the technically demanding equipment associated with d-DNP.^9,187^ Further advances in catalyst design and agent purification steps are expected to increase signal enhancements, biocompatibility, and consequently, further progress in this direction is expected. Optimisation of current catalysts is expected to improve the NMR signal enhancements that can be achieved, which in turn facilitates MRI detection of molecules at lower concentrations. Continued development of novel catalysts that can break the symmetry of para-H_2_ is likely to stimulate hyperpolarisation of a greater variety of metabolic imaging probes for disease diagnosis.

### 8.2 Hyperpolarised metal dihydride catalysts for coligand sensing: applications for mixture analysis by NMR

A selection of PHIP catalysts have been engineered with reporter hydride NMR signals designed to act as an indirect marker of a target analyte bound to the metal centre. For example, [IrCl(CO)(PPh_3_)_2_] can activate para-H_2_ to form [IrCl(H)_2_(CO)(PPh_3_)_2_] which can be detected with enhanced hydride NMR signals.^46^ Reaction of this catalyst with the analytes pyridine, benzimidazole, purine, or adenine, forms complexes of the form [IrCl(H)_2_(PPh_3_)_2_(analyte)] which also display enhanced hydride NMR resonances with distinctive chemical shifts that depend on the identity of the analyte. The improved NMR sensitivity provided by PHIP allows picomolar concentrations of these analytes to be detected *via* hyperpolarised hydride signals.

Similar effects can be achieved using SABRE catalysts, which also display hydride NMR chemical shifts sensitive to the identity of the substrate molecule *trans* to them.^38,51^ In particular, catalysts with inequivalent hydride ligands have been highly beneficial as their NMR signals display a wider chemical shift dispersion in a spectrally uncrowded region.^51^ The enhanced hydride signals of these [Ir(H)_2_(IMes)(*N*sub_1_)(*N*sub_2_)(*N*sub_3_)]Cl catalysts have been used to detect specific analytes present in complex mixtures and has been applied to study metabolomics and other systems that contain a great variety of molecules.^50,51^ In these examples, peak overlap, even within the hydride region of ^1^H NMR spectra, can often become a problem due to the large number of SABRE-amenable analytes in the mixture. It is therefore often advantageous to use 2D NMR methods such as COSY,^188,189^ DOSY^190,191^ or others^49,51^ to provide increased chemical resolution between analytes.

**Fig. 18:**
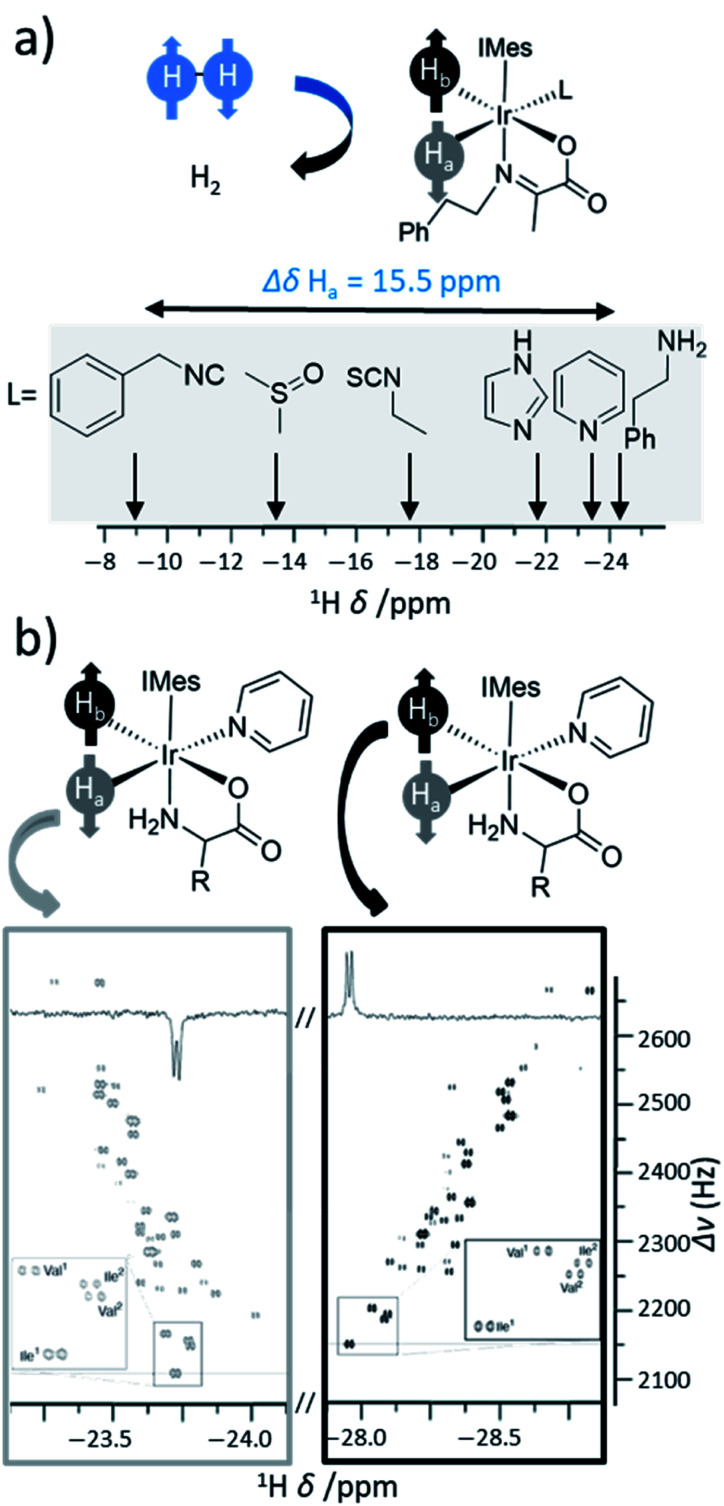
(a) The enhanced hydride NMR signals of PHIP and SABRE catalysts can provide significant chemical shift resolution which is of great use for the indirect detection of analytes in mixtures.^38^ (b) Example 2D NMR measurements that utilise PHIP hyperpolarisation of metal dihydride catalysts for sensing and identification of complex mixtures of amino acids. Adapted from ref. 49.

A further way to increase chemical shift resolution of these reporter complexes is through novel catalyst design. Recently, the enhanced hydride signals of [Ir(H)_2_(IMes)(κ^2^-*N*,*O*-imine)(sub)] catalysts have been reported as sensitive markers of particular analytes.^38^ They can achieve much wider chemical shift dispersion for their hydride signals than [Ir(H)_2_(IMes)(*N*sub_1_)(*N*sub_2_)(*N*sub_3_)]Cl systems. The chemical inequivalence of these hydrides, which are located *trans* to the *O*-donor site of the imine and *trans* to the analyte which can bind through an N-, S- or even C-donor site (Fig. 18a) is advantageous in preventing NMR signal overlap.^37,38^ As a consequence, the hydride resonance *trans* to the analyte can display signals over a 15.5 ppm chemical shift range depending on the identity of the analyte bound within the catalyst.^38^ Aliphatic and aromatic NMR signals of the analyte itself can become enhanced *via* SABRE, but for complex mixtures these signals are poorly resolved. The enhanced hydride signals of closely related [Ir(H)_2_(IMes)(κ^2^-*N*,*O*-amino acids)(pyridine)] catalysts have been used to distinguish different amino acids (Fig. 18b) in complex bio-mixtures.^49^ The applications of these catalysts in NMR mixture analysis and biofluid analysis has been clearly demonstrated and further examples are expected in the years ahead.

## 9. Conclusions

The use of para-H_2_ to enhance NMR signal intensity is a versatile platform that allows the hyperpolarisation of a range of different functionalities including metal complexes,^14,30,36,38,45^ alkenes,^14,30,57^ alkanes,^14,30^ esters,^88^ aldehydes,^23–27,114^ carboxylic acids,^126,192^ N-heterocycles,^17,29^ amines,^58,193^ diazirines,^124,194,195^ nitriles,^167^ silanes,^132^ sulfoxides,^147,172^ α-ketoacids,^125,146,147,157,196^ amino acids,^197^ peptides^78,80,82,198^ and many others. Enhanced NMR signals can be achieved most commonly for ^1^H, ^13^C and ^15^N sites, although PHIP and SABRE-enhanced ^2^H,^199,200^^11^B,^28,64,66^^19^F,^85,128,129,201^^29^Si,^131,132^^31^P,^39,69,74,130^ and ^119^Sn^131^ NMR signals have also been reported. The preparation of these molecules in a hyperpolarised state allows exciting applications in the areas of mechanistic elucidations,^11^ reaction monitoring,^139,196,202^ mixture analysis,^51,188,189,203,204^ biomedical imaging,^6,107,182,205,206^ and many others. In recent years this list of applications has even grown to include novel uses in magnetooptics,^207^ RASER physics,^208^ micron-scale NMR,^209^ SQUID-based NMR,^210^ and zero-field NMR.^211,212^

Development of novel catalyst systems has spurred some of the greatest advances in this area of science by expanding the scope of molecules that can be hyperpolarised. Many other advances in hyperpolarisation are occurring simultaneously: proton exchange effects have allowed polarisation to be relayed to molecules that might not contain any unsaturated (PHIP-X)^213^ or iridium ligating (SABRE-Relay)^214^ sites such as alcohols,^215^ sugars,^216^ silanols^132^ and many others.^213,214,217^ It is expected that further catalyst development will be a large driving force behind further extension of substrate scope, increased efficiency, and enhanced biocompatibility. In particular, novel metal-free,^28,64–67^*trans* hydrogenation,^106,109,181,182^ sulfoxide-containing SABRE catalysts^125,146,147,157^ and cobalt-based SABRE-like systems^59,179^ are likely to lead to exciting new results in this area in the years ahead.

## Abbreviations

ALTADENA: Adiabatic longitudinal transport after dissociation engenders nuclear alignment
BINAP: 2,20-Bis(diphenylphosphino)-1,10-binaphthyl
CEST: Chemical exchange saturation transfer
COD: *cis*,*cis*-1,5-Cyclooctadiene
COSY: Correlation spectroscopy
Cp*: 1,2,3,4,5-Pentamethylcyclopentadiene
Cy: Cyclohexyl
dppb: 1,4-Bis(diphenylphosphino)butane
dppe: (Diphenylphosphino)ethane
d-DNP: Dissolution dynamic nuclear polarisation
DFT: Density functional theory
DOSY: Diffusion ordered spectroscopy
EL-4: A cancer cell line
Et: Ethyl
EXSY: Exchange spectroscopy
FLP: Frustrated Lewis pair
*gem*
: *Geminal*
HCAT: Hydroborane catalyst *N*,*N*-dimethyl-2-[(pentafluorophenyl)boryl]aniline
HP: Hyperpolarised
*I*
: Nuclear spin quantum number
IMes: 1,3-Bis(2,4,6-trimethylphenyl)imidazole-2-ylidene
INEPT: Insensitive nuclei enhancement by polarisation transfer
*J*
: *J* coupling constant
Me: Methyl
Mes: Mesityl
^Mes^CCC: Bis(mesityl-benzimidazol-2-ylidene)phenyl
MFC: Magnetic field cycling
NHC: N-Heterocyclic carbene
NMR: Nuclear magnetic resonance
O-Sub: O-donor substrate
OneH: One proton
OPSY: Only parahydrogen spectroscopy
Ortho-H_2_: Orthohydrogen
Para-H_2_: Parahydrogen
PASADENA: Parahydrogen and synthesis allow dramatically enhanced nuclear alignment
Ph: Phenyl
PHIP: Parahydrogen induced polarisation
PHIP-IE: PHIP insertion elimination
PHIP-X: PHIP exchange
Phox: 2-(2-(Diphenylphosphanyl)phenyl)-4,5-dihydrooxazole
ppm: Parts per million
PTF: Polarisation transfer field
Py: Pyridine
QCAT: 1-{2-[Bis(pentafluorophenyl)boryl]benzyl}-2,2,4,7-tetramethyl-1,2,3,4-tetrahydroquinoline
RASER: Radio-frequency amplification by stimulated emission of radiation
RF: Radiofrequency
SABRE: Signal amplification by reversible exchange
SEOP: Spin exchange optical pumping
SQUID: Superconducting quantum interference device
TEP: Tolman electronic parameter
Tf: Triflate
THF: Tetrahydrofuran
THP: Tris(hydroxymethyl)phosphine
*T*_1_
: Longitudinal relaxation time
^*t*^Bu: Tertiary butyl
*T*_LLS_
: Relaxation time of long lived singlet order
Xantphos: 4,5-Bis(diphenylphosphino)-9,9-dimethylxanthene

## Author contributions

BJT: conceptualisation, writing – original draft, review and editing. VVZ: conceptualisation, writing – review and editing.

## Conflicts of interest

There are no conflicts to declare.

## Supplementary Material

SC-013-D2SC00737A-s001

## References

[cit1] Keeler J. , *Understanding NMR Spectroscopy*, John Wiley & Sons, Chichester, 2010

[cit2] Hyperpolarization Methods in NMR Spectroscopy, Topics in Current Chemistry, ed. L. T. Kuhn, Springer, Berlin, 2013, vol. 338

[cit3] Ardenkjaer-Larsen J., Boebinger G. S., Comment A., Duckett S., Edison A. S., Engelke F., Griesinger C., Griffin R. G., Hilty C., Maeda H., Parigi G., Prisner T., Ravera E., van Bentum J., Vega S., Webb A., Luchinat C., Schwalbe H., Frydman L. (2015). Angew. Chem., Int. Ed..

[cit4] Nikolaou P., Goodson B. M., Chekmenev E. Y. (2015). Chem.–Eur. J..

[cit5] Kovtunov K. V., Pokochueva E. V., Salnikov O. G., Cousin S. F., Kurzbach D., Vuichoud B., Jannin S., Chekmenev E. Y., Goodson B. M., Barskiy D. A. (2018). Chem.–Asian J..

[cit6] Hövener J., Pravdivtsev A. N., Kidd B., Bowers C. R., Glöggler S., Kovtunov K. V., Plaumann M., Katz-Brull R., Buckenmaier K., Jerschow A., Reineri F., Theis T., Shchepin R. V., Wagner S., Bhattacharya P., Zacharias N. M., Chekmenev E. Y. (2018). Angew. Chem., Int. Ed..

[cit7] Duckett S. B., Wood N. J. (2008). Coord. Chem. Rev..

[cit8] Walker T. G., Happer W. (1997). Rev. Mod. Phys..

[cit9] Keshari K. R., Wilson D. M. (2014). Chem. Soc. Rev..

[cit10] Eisenberg R. (1991). Acc. Chem. Res..

[cit11] Duckett S. B., Blazina D. (2003). Eur. J. Inorg. Chem..

[cit12] Duckett S. B., Mewis R. E. (2012). Acc. Chem. Res..

[cit13] Schmidt A. B., Bowers C. R., Buckenmaier K., Chekmenev E. Y., de Maissin H., Eills J., Ellermann F., Glöggler S., Gordon J. W., Knecht S., Koptyug I. V., Kuhn J., Pravdivtsev A. N., Reineri F., Theis T., Therm K., Hövener J.-B. (2022). Anal. Chem..

[cit14] Bowers C. R., Weitekamp D. P. (1987). J. Am. Chem. Soc..

[cit15] Kirss R. U., Eisenschmid T. C., Eisenberg R. (1988). J. Am. Chem. Soc..

[cit16] Rayner P. J., Duckett S. (2018). Angew. Chem., Int. Ed..

[cit17] Barskiy D. A., Knecht S., Yurkovskaya A. V., Ivanov K. L. (2019). Prog. Nucl. Magn. Reson. Spectrosc..

[cit18] Canet D., Aroulanda C., Mutzenhardt P., Aime S., Gobetto R., Reineri F. (2006). Concepts Magn. Reson., Part A.

[cit19] Adams R. W., Duckett S. B., Green R. A., Williamson D. C., Green G. G. R. (2009). J. Chem. Phys..

[cit20] Birchall J. R., Coffey A. M., Goodson B. M., Chekmenev E. Y. (2020). Anal. Chem..

[cit21] Feng B., Coffey A. M., Colon R. D., Chekmenev E. Y., Waddell K. W. (2012). J. Magn. Reson..

[cit22] Ellermann F., Pravdivtsev A., Hövener J.-B. (2021). Magn. Reson..

[cit23] Permin A. B., Eisenberg R. (2002). J. Am. Chem. Soc..

[cit24] Fox D. J., Duckett S. B., Flaschenriem C., Brennessel W. W., Schneider J., Gunay A., Eisenberg R. (2006). Inorg. Chem..

[cit25] Guan D., Godard C., Polas S. M., Tooze R. P., Whitwood A. C., Duckett S. B. (2019). Dalton Trans..

[cit26] Godard C., Duckett S. B., Polas S., Tooze R., Whitwood A. C. (2009). Dalton Trans..

[cit27] Godard C., Duckett S. B., Polas S., Tooze R., Whitwood A. C. (2005). J. Am. Chem. Soc..

[cit28] Zakharov D. O., Chernichenko K., Sorochkina K., Yang S., Telkki V.-V., Repo T., Zhivonitko V. V. (2021). Chem.–Eur. J..

[cit29] Adams R. W., Aguilar J. A., Atkinson K. D., Cowley M. J., Elliott P. I. P., Duckett S. B., Green G. G. R., Khazal I. G., López-Serrano J., Williamson D. C. (2009). Science.

[cit30] Bowers C. R., Weitekamp D. P. (1986). Phys. Rev. Lett..

[cit31] Eisenschmid T. C., Kirss R. U., Deutsch P. P., Hommeltoft S. I., Eisenberg R., Bargon J., Lawler R. G., Balch A. L. (1987). J. Am. Chem. Soc..

[cit32] Pravica M. G., Weitekamp D. P. (1988). Chem. Phys. Lett..

[cit33] Kireev N. V., Kiryutin A. S., Pavlov A. A., Yurkovskaya A. V., Musina E. I., Karasik A. A., Shubina E. S., Ivanov K. L., Belkova N. V. (2021). Eur. J. Inorg. Chem..

[cit34] Kiryutin A. S., Sauer G., Yurkovskaya A. V., Limbach H.-H., Ivanov K. L., Buntkowsky G. (2017). J. Phys. Chem. C.

[cit35] Knecht S., Hadjiali S., Barskiy D. A., Pines A., Sauer G., Kiryutin A. S., Ivanov K. L., Yurkovskaya A. V., Buntkowsky G. (2019). J. Phys. Chem. C.

[cit36] Procacci B., Aguiar P. M., Halse M. E., Perutz R. N., Duckett S. B. (2016). Chem. Sci..

[cit37] Tickner B. J., Iali W., Roy S. S., Whitwood A. C., Duckett S. B. (2019). ChemPhysChem.

[cit38] Tickner B. J., John R. O., Roy S. S., Hart S. J., Whitwood A. C., Duckett S. B. (2019). Chem. Sci..

[cit39] Zhivonitko V. V., Skovpin I. V., Koptyug I. V. (2015). Chem. Commun..

[cit40] Chock P. B., Halpern J. (1966). J. Am. Chem. Soc..

[cit41] Halpern J. (1959). J. Phys. Chem..

[cit42] Kirss R. U., Eisenberg R. (1989). J. Organomet. Chem..

[cit43] Bargon J. , in The Handbook of Homogeneous Hydrogenation, ed. J. G. de Vries and C. J. Elsevier, Wiley, Weinheim, 2006, pp. 313–358

[cit44] Ahlquist M., Gustafsson M., Karlsson M., Thaning M., Axelsson O., Wendt O. F. (2007). Inorg. Chim. Acta.

[cit45] Duckett S. B., Newell C. L., Eisenberg R. (1994). J. Am. Chem. Soc..

[cit46] Wood N. J., Brannigan J. A., Duckett S. B., Heath S. L., Wagstaff J. (2007). J. Am. Chem. Soc..

[cit47] López-Serrano J., Duckett S. B., Lledós A. (2006). J. Am. Chem. Soc..

[cit48] Lloyd L. S., Asghar A., Burns M. J., Charlton A., Coombes S., Cowley M. J., Dear G. J., Duckett S. B., Genov G. R., Green G. G. R. (2014). Catal. Sci. Technol..

[cit49] Sellies L., Aspers R., Feiters M. C., Rutjes F., Tessari M. (2021). Angew. Chem..

[cit50] Reimets N., Ausmees K., Vija S., Reile I. (2021). Anal. Chem..

[cit51] Sellies L., Reile I., Aspers R. L. E. G., Feiters M. C., Rutjes F. P. J. T., Tessari M. (2019). Chem. Commun..

[cit52] Procacci B., Aguiar P. M., Halse M. E., Perutz R. N., Duckett S. B. (2016). Chem. Sci..

[cit53] Harthun A., Giernoth R., Elsevier C. J., Bargon J. (1996). Chem. Commun..

[cit54] López-Serrano J., Duckett S. B., Dunne J. P., Godard C., Whitwood A. C. (2008). Dalton Trans..

[cit55] Guan D., Holmes A. J., López-Serrano J., Duckett S. B. (2017). Catal. Sci. Technol..

[cit56] McCormick J., Grunfeld A. M., Ertas Y. N., Biswas A. N., Marsh K. L., Wagner S., Glöggler S., Bouchard L.-S. (2017). Anal. Chem..

[cit57] Muhammad S. R., Greer R. B., Ramirez S. B., Goodson B. M., Fout A. R. (2021). ACS Catal..

[cit58] Tokmic K., Jackson B. J., Salazar A., Woods T. J., Fout A. R. (2017). J. Am. Chem. Soc..

[cit59] Tokmic K., Greer R. B., Zhu L., Fout A. R. (2018). J. Am. Chem. Soc..

[cit60] Koptyug I. V., Kovtunov K. V., Burt S. R., Anwar M. S., Hilty C., Han S.-I., Pines A., Sagdeev R. Z. (2007). J. Am. Chem. Soc..

[cit61] Pokochueva E. V., Burueva D. B., Salnikov O. G., Koptyug I. V. (2021). ChemPhysChem.

[cit62] Kovtunov K. V., Salnikov O. G., Skovpin I. V., Chukanov N. V., Burueva D. B., Koptyug I. V. (2020). Pure Appl. Chem..

[cit63] Frustrated Lewis Pairs I: Uncovering and Understanding, Topics in Current Chemistry, ed. G. Erker and D. W. Stephan, Springer, Berlin, 2013, vol. 332

[cit64] Sorochkina K., Zhivonitko V. V., Chernichenko K., Telkki V.-V., Repo T., Koptyug I. V. (2018). J. Phys. Chem. Lett..

[cit65] Zhivonitko V. V., Sorochkina K., Chernichenko K., Kótai B., Földes T., Pápai I., Telkki V.-V., Repo T., Koptyug I. (2016). Phys. Chem. Chem. Phys..

[cit66] Zhivonitko V. V., Telkki V.-V., Chernichenko K., Repo T., Leskelä M., Sumerin V., Koptyug I. V. (2014). J. Am. Chem. Soc..

[cit67] Zhivonitko V. V., Bresien J., Schulz A., Koptyug I. V. (2019). Phys. Chem. Chem. Phys..

[cit68] Longobardi L. E., Russell C. A., Green M., Townsend N. S., Wang K., Holmes A. J., Duckett S. B., McGrady J. E., Stephan D. W. (2014). J. Am. Chem. Soc..

[cit69] Zhivonitko V. V., Beer H., Zakharov D. O., Bresien J., Schulz A. (2021). ChemPhysChem.

[cit70] Holtkamp P., Schwabedissen J., Neumann B., Stammler G., Koptyug I., Zhivonitko V., Mitzel N. W. (2020). Chem.–Eur. J..

[cit71] Dedieu A., Humbel S., Elsevier C., Grauffel C. (2004). Theor. Chem. Acc..

[cit72] Albietz P. J., Houlis J. F., Eisenberg R. (2002). Inorg. Chem..

[cit73] Papp G., Horváth H., Joó F. (2019). ChemCatChem.

[cit74] Eisenschmid T. C., McDonald J., Eisenberg R., Lawler R. G. (1989). J. Am. Chem. Soc..

[cit75] Giernoth R., Huebler P., Bargon J. (1998). Angew. Chem., Int. Ed..

[cit76] Glöggler S., Wagner S., Bouchard L.-S. (2015). Chem. Sci..

[cit77] Körner M., Sauer G., Heil A., Nasu D., Empting M., Tietze D., Voigt S., Weidler H., Gutmann T., Avrutina O. (2013). Chem. Commun..

[cit78] Sauer G., Nasu D., Tietze D., Gutmann T., Englert S., Avrutina O., Kolmar H., Buntkowsky G. (2014). Angew. Chem., Int. Ed..

[cit79] Kiryutin A. S., Sauer G., Tietze D., Brodrecht M., Knecht S., Yurkovskaya A. V., Ivanov K. L., Avrutina O., Kolmar H., Buntkowsky G. (2019). Chem.–Eur. J..

[cit80] Gruppi F., Xu X., Zhang B., Tang J. A., Jerschow A., Canary J. W. (2012). Angew. Chem..

[cit81] Mandal R., Pham P., Hilty C. (2021). Chem. Sci..

[cit82] Ratajczyk T., Buntkowsky G., Gutmann T., Fedorczyk B., Mames A., Pietrzak M., Szkudlarek P., Puzio Z. (2021). ChemBioChem.

[cit83] Duckett S. B., Newell C. L., Eisenberg R. (1993). J. Am. Chem. Soc..

[cit84] Kozinenko V. P., Kiryutin A. S., Yurkovskaya A. V., Ivanov K. L. (2019). J. Magn. Reson..

[cit85] Kuhn L. T., Bommerich U., Bargon J. (2006). J. Phys. Chem. A.

[cit86] Stephan M., Kohlmann O., Niessen H. G., Eichhorn A., Bargon J. (2002). Magn. Reson. Chem..

[cit87] Jóhannesson H., Axelsson O., Karlsson M. (2004). C. R. Phys..

[cit88] Cavallari E., Carrera C., Boi T., Aime S., Reineri F. (2015). J. Phys. Chem. B.

[cit89] Joalland B., Schmidt A. B., Kabir M. S. H., Chukanov N. V., Kovtunov K. V., Koptyug I. V., Hennig J., Hövener J.-B., Chekmenev E. Y. (2019). Anal. Chem..

[cit90] Itoda M., Naganawa Y., Ito M., Nonaka H., Sando S. (2019). RSC Adv..

[cit91] Shchepin R. V., Barskiy D. A., Coffey A. M., Manzanera Esteve I. V., Chekmenev E. Y. (2016). Angew. Chem..

[cit92] Emondts M., Colell J. F. P., Blümich B., Schleker P. P. M. (2017). Phys. Chem. Chem. Phys..

[cit93] Jessop P. G., Morris R. H. (1992). Coord. Chem. Rev..

[cit94] Sánchez-Delgado R. A., Rosales M. (2000). Coord. Chem. Rev..

[cit95] Gridnev I. D., Higashi N., Asakura K., Imamoto T. (2000). J. Am. Chem. Soc..

[cit96] Bondar O., Cavallari E., Carrera C., Aime S., Reineri F. (2021). Catal. Today.

[cit97] Cavallari E., Carrera C., Aime S., Reineri F. (2018). J. Magn. Reson..

[cit98] Shchepin R. V., Coffey A. M., Waddell K. W., Chekmenev E. Y. (2012). J. Phys. Chem. Lett..

[cit99] Zhivonitko V. V., Skovpin I. V., Szeto K. C., Taoufik M., Koptyug I. V. (2018). J.
Phys. Chem. C.

[cit100] Burueva D. B., Smirnov A. A., Bulavchenko O. A., Prosvirin I. P., Gerasimov E. Y., Yakovlev V. A., Kovtunov K. V., Koptyug I. V. (2020). Top. Catal..

[cit101] Zhao E. W., Zheng H., Zhou R., Hagelin-Weaver H. E., Bowers C. R. (2015). Angew. Chem..

[cit102] Johnson C. E., Eisenberg R. (1985). J. Am. Chem. Soc..

[cit103] Tokmic K., Fout A. R. (2016). J. Am. Chem. Soc..

[cit104] Schleyer D., Niessen H. G., Bargon J. (2001). New J. Chem..

[cit105] Leutzsch M., Wolf L. M., Gupta P., Fuchs M., Thiel W., Farès C., Fürstner A. (2015). Angew. Chem..

[cit106] Guthertz A., Leutzsch M., Wolf L. M., Gupta P., Rummelt S. M., Goddard R., Farès C., Thiel W., Fürstner A. (2018). J. Am. Chem. Soc..

[cit107] Stewart N. J., Nakano H., Sugai S., Tomohiro M., Kase Y., Uchio Y., Yamaguchi T., Matsuo Y., Naganuma T., Takeda N., Nishimura I., Hirata H., Hashimoto T., Matsumoto S. (2021). ChemPhysChem.

[cit108] Biberger T., Hess S. N., Leutzsch M., Fürstner A. (2022). Angew. Chem..

[cit109] Dagys L., Ripka B., Leutzsch M., Moustafa G. A. I., Eills J., Colell J. F. P., Levitt M. H. (2020). Magn. Reson..

[cit110] Harthun A., Selke R., Bargon J. (1996). Angew. Chem., Int. Ed..

[cit111] Skovpin I. V., Zhivonitko V. V., Koptyug I. V. (2011). Appl. Magn. Reson..

[cit112] Pravdivtsev A. N., Brahms A., Kienitz S., Sönnichsen F. D., Hövener J., Herges R. (2021). ChemPhysChem.

[cit113] Lehmkuhl S., Emondts M., Schubert L., Spannring P., Klankermayer J., Bluemich B., Schleker P. (2017). ChemPhysChem.

[cit114] Godard C., Duckett S. B., Henry C., Polas S., Toose R., Whitwood A. C. (2004). Chem. Commun..

[cit115] Pravdivtsev A. N., Ivanov K. L., Yurkovskaya A. V., Petrov P. A., Limbach H.-H., Kaptein R., Vieth H.-M. (2015). J. Magn. Reson..

[cit116] Pravdivtsev A. N., Yurkovskaya A. V., Vieth H., Ivanov K. L., Kaptein R. (2013). ChemPhysChem.

[cit117] Eshuis N., Aspers R. L. E. G., van Weerdenburg B. J. A., Feiters M. C., Rutjes F. P. J. T., Wijmenga S. S., Tessari M. (2016). J. Magn. Reson..

[cit118] Dücker E. B., Kuhn L. T., Münnemann K., Griesinger C. (2012). J. Magn. Reson..

[cit119] Kiryutin A. S., Yurkovskaya A. V., Zimmermann H., Vieth H., Ivanov K. L. (2018). Magn. Reson. Chem..

[cit120] Shchepin R. V., Barskiy D. A., Coffey A. M., Theis T., Shi F., Warren W. S., Goodson B. M., Chekmenev E. Y. (2016). ACS Sens..

[cit121] Theis T., Truong M. L., Coffey A. M., Shchepin R. V., Waddell K. W., Shi F., Goodson B. M., Warren W. S., Chekmenev E. Y. (2015). J. Am. Chem. Soc..

[cit122] Truong M. L., Theis T., Coffey A. M., Shchepin R. V., Waddell K. W., Shi F., Goodson B. M., Warren W. S., Chekmenev E. Y. (2015). J. Phys. Chem. C.

[cit123] Fekete M., Ahwal F., Duckett S. B. (2020). J. Phys. Chem. B.

[cit124] Shen K., Logan A. W. J., Colell J. F. P., Bae J., Ortiz Jr G. X., Theis T., Warren W. S., Malcolmson S. J., Wang Q. (2017). Angew. Chem..

[cit125] Iali W., Roy S. S., Tickner B. J., Ahwal F., Kennerley A. J., Duckett S. B. (2019). Angew. Chem..

[cit126] Gemeinhardt M. E., Limbach M. N., Gebhardt T. R., Eriksson C. W., Eriksson S. L., Lindale J. R., Goodson E. A., Warren W. S., Chekmenev E. Y., Goodson B. M. (2020). Angew. Chem..

[cit127] Zhou Z., Yu J., Colell J. F. P., Laasner R., Logan A., Barskiy D. A., Shchepin R. V., Chekmenev E. Y., Blum V., Warren W. S. (2017). J. Phys. Chem. Lett..

[cit128] Shchepin R. V., Goodson B. M., Theis T., Warren W. S., Chekmenev E. Y. (2017). ChemPhysChem.

[cit129] Olaru A. M., Robertson T. B. R., Lewis J. S., Antony A., Iali W., Mewis R. E., Duckett S. B. (2018). ChemistryOpen.

[cit130] Burns M. J., Rayner P. J., Green G. G. R., Highton L. A. R., Mewis R. E., Duckett S. B. (2015). J. Phys. Chem. B.

[cit131] Olaru A. M., Burt A., Rayner P. J., Hart S. J., Whitwood A. C., Green G. G. R., Duckett S. B. (2016). Chem. Commun..

[cit132] Rayner P. J., Richardson P. M., Duckett S. B. (2020). Angew. Chem..

[cit133] Barskiy D. A., Kovtunov K. V., Koptyug I. V., He P., Groome K. A., Best Q. A., Shi F., Goodson B. M., V Shchepin R., Coffey A. M. (2014). J. Am. Chem. Soc..

[cit134] Theis T., Truong M., Coffey A. M., Chekmenev E. Y., Warren W. S. (2014). J. Magn. Reson..

[cit135] Lindale J. R., Tanner C. P. N., Eriksson S. L., Warren W. S. (2019). J. Magn. Reson..

[cit136] Pravdivtsev A. N., Yurkovskaya A. V., Vieth H.-M., Ivanov K. L. (2015). J. Phys. Chem. B.

[cit137] Knecht S., Kiryutin A. S., Yurkovskaya A. V., Ivanov K. L. (2018). J. Magn. Reson..

[cit138] Knecht S., Kiryutin A. S., Yurkovskaya A. V., Ivanov K. L. (2019). Mol. Phys..

[cit139] Semenova O., Richardson P. M., Parrott A. J., Nordon A., Halse M. E., Duckett S. B. (2019). Anal. Chem..

[cit140] Atkinson K. D., Cowley M. J., Duckett S. B., Elliott P. I. P., Green G. G. R., López-Serrano J., Khazal I. G., Whitwood A. C. (2009). Inorg. Chem..

[cit141] Cowley M. J., Adams R. W., Atkinson K. D., Cockett M. C. R., Duckett S. B., Green G. G. R., Lohman J. A. B., Kerssebaum R., Kilgour D., Mewis R. E. (2011). J. Am. Chem. Soc..

[cit142] Holmes A. J., Rayner P. J., Cowley M. J., Green G. G. R., Whitwood A. C., Duckett S. B. (2015). Dalton Trans..

[cit143] Thomas A., Haake M., Grevels F., Bargon J. (1994). Angew. Chem., Int. Ed..

[cit144] Lin K., TomHon P., Lehmkuhl S., Laasner R., Theis T., Blum V. (2021). ChemPhysChem.

[cit145] Zeng H., Xu J., Gillen J., McMahon M. T., Artemov D., Tyburn J., Lohman J. A. B., Mewis R. E., Atkinson K. D., Green G. G. R., Duckett S. B., van Zijl P. C. M. (2013). J. Magn. Reson. Imag..

[cit146] Tickner B. J., Semenova O., Iali W., Rayner P. J., Whitwood A. C., Duckett S. B. (2020). Catal. Sci. Technol..

[cit147] Tickner B. J., Lewis J. S., John R. O., Whitwood A. C., Duckett S. B. (2019). Dalton Trans..

[cit148] Eriksson S. L., Lindale J. R., Li X., Warren W. S. (2022). Sci. Adv..

[cit149] Atkinson K. D., Cowley M. J., Elliott P. I. P., Duckett S. B., Green G. G. R., Lopez-Serrano J., Whitwood A. C. (2009). J. Am. Chem. Soc..

[cit150] Colell J., Logan A. W. J., Zhou Z., Lindale J. R., Laasner R., Shchepin R., Chekmenev E., Blum V., Warren W. S., Malcolmson S. J. (2020). Chem. Commun..

[cit151] Gusev D. G. (2009). Organometallics.

[cit152] Kelly III R. A., Clavier H., Giudice S., Scott N. M., Stevens E. D., Bordner J., Samardjiev I., Hoff C. D., Cavallo L., Nolan S. P. (2008). Organometallics.

[cit153] Rayner P. J., Norcott P., Appleby K. M., Iali W., John R. O., Hart S. J., Whitwood A. C., Duckett S. B. (2018). Nat. Commun..

[cit154] van Weerdenburg B. J. A., Eshuis N., Tessari M., Rutjes F. P. J. T., Feiters M. C. (2015). Dalton Trans..

[cit155] van Weerdenburg B. J. A., Glöggler S., Eshuis N., Engwerda A. H. J. T., Smits J. M. M., de Gelder R., Appelt S., Wymenga S. S., Tessari M., Feiters M. C., Blümich B., Rutjes F. P. J. T. (2013). Chem. Commun..

[cit156] Hadjiali S., Savka R., Plaumann M., Bommerich U., Bothe S., Gutmann T., Ratajczyk T., Bernarding J., Limbach H.-H., Plenio H. (2019). Appl. Magn. Reson..

[cit157] Tickner B. J., Ahwal F., Whitwood A. C., Duckett S. B. (2021). ChemPhysChem.

[cit158] Tickner B. J., Borozdina Y., Duckett S. B., Angelovski G. (2021). Dalton Trans..

[cit159] Iali W., Green G. G. R., Hart S. J., Whitwood A. C., Duckett S. B. (2016). Inorg. Chem..

[cit160] Ruddlesden A. J., Mewis R. E., Green G. G. R., Whitwood A. C., Duckett S. B. (2015). Organometallics.

[cit161] Fekete M., Gibard C., Dear G. J., Green G. G. R., Hooper A. J. J., Roberts D., Cisnetti F., Duckett S. B. (2015). Dalton Trans..

[cit162] Shi F., He P., Best Q. A., Groome K., Truong M. L., Coffey A. M., Zimay G., Shchepin R. V., Waddell K. W., Chekmenev E. Y. (2016). J. Phys. Chem. C.

[cit163] Spannring P., Reile I., Emondts M., Schleker P. P. M., Hermkens N. K. J., van der Zwaluw N. G. J., van Weerdenburg B. J. A., Tinnemans P., Tessari M., Blümich B. (2016). Chem.–Eur. J..

[cit164] Wong C. M., Fekete M., Nelson-Forde R., Gatus M. R. D., Rayner P. J., Whitwood A. C., Duckett S. B., Messerle B. A. (2018). Catal. Sci. Technol..

[cit165] Pham P., Hilty C. (2020). Chem. Commun..

[cit166] Rayner P. J., Gillions J. P., Hannibal V. D., John R. O., Duckett S. B. (2021). Chem. Sci..

[cit167] Mewis R. E., Green R. A., Cockett M. C. R., Cowley M. J., Duckett S. B., Green G. G. R., John R. O., Rayner P. J., Williamson D. C. (2015). J. Phys. Chem. B.

[cit168] Mandal R., Pham P., Hilty C. (2020). ChemPhysChem.

[cit169] Diaz-Rullo F. F., Zamberlan F., Mewis R. E., Fekete M., Broche L., Cheyne L. A., DallAngelo S., Duckett S. B., Dawson D., Zanda M. (2017). Bioorg. Med. Chem..

[cit170] Colell J. F. P., Logan A. W. J., Zhou Z., Shchepin R. V., Barskiy D. A., Ortiz Jr G. X., Wang Q., Malcolmson S. J., Chekmenev E. Y., Warren W. S. (2017). J. Phys. Chem. C.

[cit171] TomHon P., Abdulmojeed M., Adelabu I., Nantogma S., Kabir M. S. H., Lehmkuhl S., Chekmenev E. Y., Theis T. (2022). J. Am. Chem. Soc..

[cit172] Tickner B. J., Parker R. R., Whitwood A. C., Duckett S. B. (2019). Organometallics.

[cit173] Fekete M., Roy S. S., Duckett S. B. (2020). Phys. Chem. Chem. Phys..

[cit174] Barskiy D. A., Pravdivtsev A. N., Ivanov K. L., Kovtunov K. V., Koptyug I. V. (2016). Phys. Chem. Chem. Phys..

[cit175] Knecht S., Barskiy D. A., Buntkowsky G., Ivanov K. L. (2020). J. Chem. Phys..

[cit176] Theis T., Ariyasingha N. M., Shchepin R. V., Lindale J. R., Warren W. S., Chekmenev E. Y. (2018). J. Phys. Chem. Lett..

[cit177] Truong M. L., Shi F., He P., Yuan B., Plunkett K. N., Coffey A. N., Shchepin R. V., Barskiy D. A., Kovtunov K. V., Koptyug I. V., Waddell K. W., Goodson B. M., Chekmenev E. Y. (2014). J. Phys. Chem. B.

[cit178] Robertson T. B. , PhD thesis, Manchester Metropolitan University, U.K., 2019

[cit179] Tokmic K., Markus C. R., Zhu L., Fout A. R. (2016). J. Am. Chem. Soc..

[cit180] Reineri F., Cavallari E., Carrera C., Aime S. (2021). Magn. Reson. Mater. Phys., Biol. Med..

[cit181] Ripka B., Eills J., Kouřilová H., Leutzsch M., Levitt M. H., Münnemann K. (2018). Chem. Commun..

[cit182] Eills J., Cavallari E., Carrera C., Budker D., Aime S., Reineri F. (2019). J. Am. Chem. Soc..

[cit183] Knecht S., Blanchard J. W., Barskiy D., Cavallari E., Dagys L., Van Dyke E., Tsukanov M., Bliemel B., Münnemann K., Aime S., Reineri F., Levitt M. H., Buntkowsky G., Pines A., Blumler P., Budker D., Eills J. (2021). Proc. Natl. Acad. Sci..

[cit184] Wienands L., Theiß F., Eills J., Rösler L., Knecht S., Buntkowsky G. (2021). Appl. Magn. Reson..

[cit185] Witney T., Kettunen M., Hu D., Gallagher F., Bohndiek S., Napolitano R., Brindle K. (2010). Br. J. Cancer.

[cit186] Gallagher F. A., Kettunen M. I., Hu D.-E., Jensen P. R., Karlsson M., Gisselsson A., Nelson S. K., Witney T. H., Bohndiek S. E., Hansson G. (2009). Proc. Natl. Acad. Sci..

[cit187] Ardenkjær-Larsen J. H., Bowen S., Petersen J. R., Rybalko O., Vinding M. S., Ullisch M., Nielsen N. C. (2019). Magn. Reson. Med..

[cit188] Lloyd L. S., Adams R. W., Bernstein M., Coombes S., Duckett S. B., Green G. G. R., Lewis R. J., Mewis R. E., Sleigh C. J. (2012). J. Am. Chem. Soc..

[cit189] Daniele V., Legrand F., Berthault P., Dumez J., Huber G. (2015). ChemPhysChem.

[cit190] Reile I., Aspers R. L. E. G., Tyburn J., Kempf J. G., Feiters M. C., Rutjes F. P. J. T., Tessari M. (2017). Angew. Chem..

[cit191] Guduff L., Berthault P., Van Heijenoort C., Dumez J., Huber G. (2019). ChemPhysChem.

[cit192] Cavallari E., Carrera C., Aime S., Reineri F. (2017). Chem.–Eur. J..

[cit193] Iali W., Rayner P. J., Alshehri A., Holmes A. J., Ruddlesden A. J., Duckett S. B. (2018). Chem. Sci..

[cit194] Procacci B., Roy S. S., Norcott P., Turner N., Duckett S. B. (2018). J. Am. Chem. Soc..

[cit195] Theis T., Ortiz G. X., Logan A. W. J., Claytor K. E., Feng Y., Huhn W. P., Blum V., Malcolmson S. J., Chekmenev E. Y., Wang Q. (2016). Sci. Adv..

[cit196] Tickner B. J., Rayner P. J., Duckett S. B. (2020). Anal. Chem..

[cit197] Pravdivtsev A. N., Buntkowsky G., Duckett S. B., Koptyug I. V., Hövener J.-B. (2021). Angew. Chem., Int. Ed..

[cit198] Glöggler S., Müller R., Colell J., Emondts M., Dabrowski M., Blümich B., Appelt S. (2011). Phys. Chem. Chem. Phys..

[cit199] Aime S., Gobetto R., Reineri F., Canet D. (2003). J. Chem. Phys..

[cit200] Kozinenko V. P., Kiryutin A. S., Knecht S., Buntkowsky G., Vieth H.-M., Yurkovskaya A. V., Ivanov K. L. (2020). J. Chem. Phys..

[cit201] Chukanov N. V., Salnikov O. G., Shchepin R. V., Svyatova A., Kovtunov K. V., Koptyug I. V., Chekmenev E. Y. (2018). J. Phys. Chem. C.

[cit202] Chae H., Min S., Jeong H. J., Namgoong S. K., Oh S., Kim K., Jeong K. (2020). Anal. Chem..

[cit203] Eshuis N., Aspers R. L. E. G., van Weerdenburg B. J. A., Feiters M. C., Rutjes F. P. J. T., Wijmenga S. S., Tessari M. (2015). Angew. Chem., Int. Ed..

[cit204] Eshuis N., Hermkens N., van Weerdenburg B. J. A., Feiters M. C., Rutjes F. P. J. T., Wijmenga S. S., Tessari M. (2014). J. Am. Chem. Soc..

[cit205] Cavallari E., Carrera C., Aime S., Reineri F. (2019). ChemPhysChem.

[cit206] Bhattacharya P., Chekmenev E. Y., Perman W. H., Harris K. C., Lin A. P., Norton V. A., Tan C. T., Ross B. D., Weitekamp D. P. (2007). J. Magn. Reson..

[cit207] Štěpánek P., Kantola A. M. (2019). J. Phys. Chem. Lett..

[cit208] Appelt S., Lehmkuhl S., Fleischer S., Joalland B., Ariyasingha N. M., Chekmenev E. Y., Theis T. (2021). J. Magn. Reson..

[cit209] Arunkumar N., Bucher D. B., Turner M. J., TomHon P., Glenn D., Lehmkuhl S., Lukin M. D., Park H., Rosen M. S., Theis T. (2021). PRX Quantum.

[cit210] Buckenmaier K., Rudolph M., Back C., Misztal T., Bommerich U., Fehling P., Koelle D., Kleiner R., Mayer H. A., Scheffler K. (2017). Sci. Rep..

[cit211] Theis T., Ganssle P., Kervern G., Knappe S., Kitching J., Ledbetter M. P., Budker D., Pines A. (2011). Nat. Phys..

[cit212] Theis T., Ledbetter M. P., Kervern G., Blanchard J. W., Ganssle P. J., Butler M. C., Shin H. D., Budker D., Pines A. (2012). J. Am. Chem. Soc..

[cit213] Them K., Ellermann F., Pravdivtsev A. N., Salnikov O. G., Skovpin I. V., Koptyug I. V., Herges R., Hövener J.-B. (2021). J. Am. Chem. Soc..

[cit214] Iali W., Rayner P. J., Duckett S. B. (2018). Sci. Adv..

[cit215] Rayner P. J., Tickner B. J., Iali W., Fekete M., Robinson A. D., Duckett S. B. (2019). Chem. Sci..

[cit216] Richardson P. M., Iali W., Roy S. S., Rayner P. J., Halse M. E., Duckett S. B. (2019). Chem. Sci..

[cit217] Vaneeckhaute E., Tyburn J.-M., Kilgour D., Kempf J. G., Taulelle F., Martens J. A., Breynaert E. (2020). J. Phys. Chem. C.

