# Supplementary material for: Advancing homogeneous catalysis for parahydrogen-derived hyperpolarisation and its NMR applications
Source: Chem Sci. 2022 Mar 22;13(17):4670–96. doi: 10.1039/d2sc00737a (PMC9067625; doi:10.1039/d2sc00737a)
Supplement: SC-013-D2SC00737A-s001 [file SC-013-D2SC00737A-s001.pdf]

Electronic Supplementary Material (ESI) for Chemical Science.
This journal is © The Royal Society of Chemistry 2022

Supporting Information

Advancing Homogeneous Catalysis for Parahydrogen-Derived Hyperpolarisation and its NMR Applications

Ben. J. Tickner[a,b]* and Vladimir V. Zhivonitko[a]*

[a] NMR Research Unit, Faculty of Science, University of Oulu, P.O. Box 3000, 90014 Oulu, Finland.
[b] Department of Chemical and Biological Physics, Faculty of Chemistry, Weizmann Institute of Science, Rehovot, 7610001, Israel.

* Corresponding authors: ben.tickner@alumni.york.ac.uk and vladimir.zhivonitko@oulu.fi

A range of SABRE active complexes with different NHC auxiliary ligands have been reported (see Figure 12 and 13 of the manuscript) which exhibit a variety of steric and electronic properties. These can be quantified by use of % Buried Volumes (%BurV) or Tolman Electronic Parameters (TEP) respectively (Table S1). We provide here a comparison of the different %BurV and TEP parameters for IMes-derived ligands used in SABRE hyperpolarisation experiments.

Table S1: Summary of electronic and steric properties of SABRE polarisation transfer catalysts with a variety of NHC ligands.

No.	Abbreviation	R_1	R_2	R_3	R_4	R_5	TEP /cm^{-1}	BurV /%	Ref.
**5**	IMe	CH_3	H	CH_3	-	-	2054.1[¤]	26.3[¤]	[48]
**6**	Im*N*Pr$^i_2$	$CH(CH_3)_2$	H	$CH(CH_3)_2$	-	-	2050.4[%]	26.4[@]	[154],[155]
**7**	ImMe$_2$*N*Pr$^i_2$	$CH(CH_3)_2$	CH_3	$CH(CH_3)_2$	-	-	2049.6[¤]	38.4[¤]	[48]
**8**	ItBu	^{t}Bu	H	^{t}Bu	-	-	2048.9[%]	35.5[@]	[155]
**9**	Icy	Cyclohexyl	H	Cyclohexyl	-	-	2049.5[%]	27.1[@]	[154],[155]
					-	-	2049.6[¤]	27.4[¤]	[48]
**10**	Iad	Adamantyl	H	Adamantyl	-	-	2048.3[%]	36.1[@]	[155]
**11**	-	Ph	H	CH_3	-	-	-	-	[163]
**12**	-	Mesityl	H	CH_3	-	-	-	-	[163]
**13**	-	Mesityl	H	CH_2Ph	-	-	-	-	[163]
**14**	-	Mesityl	H	CH_2CH_2Ph	-	-	-	-	[163]
**15**	SIm*N*Pr$^i_2$	$CH(CH_3)_2$	H	$CH(CH_3)_2$	-	-	2050.8[%]	28.5[@]	[154],[155]
**16**	ipr-NHC	Mesityl	H	$CH(CH_3)_2$	-	-	-	-	[156]
**17**	Si-NHC	Mesityl	H	$(CH_2)_3Si(OEt)_3$	-	-	-	-	[156]
**18**	tzNHC	Mesityl	CH_3	Ph	-	-	-	-	[156]
**19**	CAAC-NHC	$CH(CH_3)_2$	-	-	-	-	-	-	[156]
**20**	IMes	CH_3	H	CH_3	H	H	2049.6[%]	31.6[@]	[154],[155]
							2049.6[#]	31.6	[153]
							2050.7[¤]	36.5[¤]	[48]
**21**	-	CH_3	H	H	H	H	2050.5[#]	30.5	[153]
**22**	-	H	H	CH_3	H	H	2050.6[#]	31.6	[153]
**23**	-	H	H	CH_2CH_3	H	H	2049.8[#]	31.7	[153]
**24**	IPr	H	H	$CH(CH_3)_2$	H	H	2050.1[#]	32.6	[153]
							2050.2[%]	33.6[@]	[154],[155]
							2051.5[¤]	44.5[¤]	[48]
**25**	-	H	CH_3	H	H	H	2049.6[#]	30.5	[153]
**26**	-	CH_3	CH_3	H	H	H	2048.9[#]	30.5	[153]
**27**	-	F	H	CH_3	H	H	2051.4[#]	31.7	[153]
**28**	-	Cl	H	CH_3	H	H	2051.7[#]	31.7	[153]
**29**	-	Br	H	CH_3	H	H	2051.9[#]	31.7	[153]
**30**	-	I	H	CH_3	H	H	2051.9[#]	31.7	[153]
**31**	-	CO_2Me	H	CH_3	H	H	2052.2[#]	31.8	[153]
**32**	-	OTf	H	CH_3	H	H	2053.2[#]	31.8	[153]
**33**	-	OAc	H	CH_3	H	H	2051.3[#]	31.7	[153]
**34**	-	Ph	H	CH_3	H	H	2050.4[#]	31.7	[153]
**35**	-	^{t}Bu	H	CH_3	H	H	2049.6[#]	31.7	[153]
**36**	-	OMe	H	CH_3	H	H	2049.3[#]	31.5	[153]
**37**	-	NMe_2	H	CH_3	H	H	2047.6[#]	31.6	[153]
**38**	-	CH_3	H	CH_3	CH_3	CH_3	2047.2[#]	31.4	[153]
**39**	-	CH_3	H	CH_3	CH_2CH_3	CH_2CH_3	2047.8[#]	31.6	[153]
**40**	-	CH_3	H	CH_3	Cl	Cl	2052.8[#]	31.7	[153]
**41**	-	CH_3	H	CH_3	Br	Br	2052.4[#]	31.8	[153]
**42**	-	Cl	H	CH_3	Cl	Cl	2054.9[#]	31.8	[153]
**43**	-	H	H	CH(Et)$_2$	H	H	2048.3[%]	-	[154]
**44**	-	CH_3	H	CH_3	NMe_2	H	-	-	[212]
**45**	SIMes	CH_3	H	CH_3	H	H	2050.8[%]	32.7[@]	[154],[155]
							2051.5[¤]	36.9[¤]	[48]
**46**	SIPr	H	H	$CH(CH_3)_2$	H	H	2051.1[%]	35.7[@]	[154],[155]
							2052.2[¤]	47.0[¤]	[48]
**47-51**	-	-	-		-	-	-	-	[161]-[163]

[¤]These values were calculated using density functional theory involving $[Ni(CO)_3(NHC)]$ and were originally reported in Ref. no. [151]

[%]Ref. no. [154] presented average infrared carbonyl stretching frequencies ($\nu_{CO,av}$) of $[Ir(NHC)(CO)_2Cl]$. TEP values were calculated from these in our work according to the established equation TEP = $0.847\nu_{CO,av} + 336$ consistent with other works.[152,153]

[@]These values were calculated in Ref. no. [155] from X-ray crystal structures of $[Ir(NHC)(CO)_2Cl]$ reported in Ref. no. [152].

[#]Values determined from average infrared carbonyl stretching frequencies ($\nu_{CO,av}$) of $[Ir(NHC)(CO)_2Cl]$

Note that reference numbers correspond to those that appear in the main manuscript
